# Viable Mice with Extensive Gene Humanization (25-kbp) Created Using Embryonic Stem Cell/Blastocyst and CRISPR/Zygote Injection Approaches

**DOI:** 10.1038/s41598-018-33408-9

**Published:** 2018-10-09

**Authors:** Tiffany Leidy-Davis, Kai Cheng, Leslie O. Goodwin, Judith L. Morgan, Wen Chun Juan, Xavier Roca, S. Tiong Ong, David E. Bergstrom

**Affiliations:** 1 Genetic Resource Science, The Jackson Laboratory, Bar Harbor, ME USA; 2 Institute of Molecular and Cell Biology, Agency for Science, Technology and Research (A*STAR), Singapore, Republic of Singapore; 3 School of Biological Sciences, Nanyang Technological University, Singapore, Republic of Singapore; 4 Cancer and Stem Cell Biology Signature Research Programme, Duke-NUS Medical School, Singapore, Republic of Singapore; 5 Department of Haematology, Singapore General Hospital, Singapore, Republic of Singapore; 6 Department of Medical Oncology, National Cancer Centre, Singapore, Republic of Singapore; 7 Department of Medicine, Duke University Medical Center, Durham, NC USA; 8 Cancer Center, The Jackson Laboratory, Bar Harbor, ME USA; 9 Present Address: Genetically Engineered Models and Services, Charles River Laboratories, Wilmington, USA; 10 Present Address: Center for Biometric Analysis, The Jackson Laboratory, Bar Harbor, USA; 11 Present Address: MSD Pharma (Singapore) Private Limited, Singapore, Republic of Singapore

## Abstract

Here, we describe an expansion of the typical DNA size limitations associated with CRISPR knock-in technology, more specifically, the physical extent to which mouse genomic DNA can be replaced with donor (in this case, human) DNA at an orthologous locus by zygotic injection. Driving our efforts was the desire to create a whole animal model that would replace 17 kilobase pairs (kbp) of the mouse *Bcl2l11* gene with the corresponding 25-kbp segment of human *BCL2L11*, including a conditionally removable segment (2.9-kbp) of intron 2, a cryptic human exon immediately 3′ of this, and a native human exon some 20 kbp downstream. Using two methods, we first carried out the replacement by employing a combination of bacterial artificial chromosome recombineering, classic embryonic stem cell (ESC) targeting, dual selection, and recombinase-driven cassette removal (ESC/Blastocyst Approach). Using a unique second method, we employed the same vector (devoid of its selectable marker cassettes), microinjecting it along with redundant single guide RNAs (sgRNAs) and *Cas9* mRNA into mouse zygotes (CRISPR/Zygote Approach). In both instances, we were able to achieve humanization of *Bcl2l11* to the extent designed, remove all selection cassettes, and demonstrate the functionality of the conditionally removable, *lox*P-flanked, 2.9-kbp intronic segment.

## Introduction

The discovery of clustered regularly interspaced short palindromic repeat (CRISPR) systems, the elucidation of their function, and their exploitation as genome engineering tools are revolutionizing genetic engineering^1–5^. Discovered as a form of adaptive immunity in bacteria and archaea, CRISPR systems consist of a series of DNA spacer elements derived from invading plasmids or viruses. Interdigitated among the spacers is a series of direct repeats. Depending on the particular system, these series are transcribed and processed into single spacer/repeat units called crRNAs (CRISPR RNAs). In turn, these crRNAs may interact with other short RNAs (*e*.*g*., tracrRNA) and one or more CRISPR-associated (Cas) proteins (*e*.*g*., CAS9 of *Streptococcus pyogenes*), culminating in the assembly of an RNA-guided endonuclease directed at degrading DNA from the offending plasmid or virus.

As genome engineering tools, the CRISPR-Cas endonucleases serve as instruments for generating DNA double-strand breaks (DSBs) with locus-of-interest specificity, at high frequency, and across a wide variety of strains and organisms^6^. When faced with DSBs, cells of the organism being perturbed may respond with a number of DNA repair pathways including the non-homologous end joining (NHEJ) pathway and the homology-directed repair (HDR) pathway^7–9^. DNA DSBs repaired by the efficient but error-prone NHEJ pathway are characterized by the deletion or insertion of a small number of nucleotides. As one might expect, these insertion/deletion events (INDELS), within the open reading frame of a protein of interest, may lead to the deletion of one or more endogenous amino acids, the insertion of one or more non-native amino acids, premature termination, or frameshift mutations. In each of these instances the modified mutant locus will commonly encode a hypomorphic or null allele of the original gene of interest.

In contrast, DSBs repaired in the presence of a homologous template (*e*.*g*., sister chromatid, donor molecule) may be repaired by homologous recombination (HR; a type of HDR)^10^. For genetic engineers, this provides the opportunity to introduce precise DNA modifications, created at the laboratory bench, into the organism under investigation, at the site of the DSB.

For classical gene-targeting, of the sort in use in the mouse for the past thirty years^11–15^, the typical paradigm, based on a large body of literature, has been to create plasmid vectors with two homology arms of a few to several kilobase pairs in length to act as donor molecules^16,17^. These arms are situated within the plasmid so as to flank investigator-altered sequences that will be incorporated into the genome after introduction of the plasmid vector into embryonic stem cells (ESCs) and homologous recombination. Positive and negative selection cassettes are frequently employed to aid in selecting the rare ESC clones containing properly integrated sequences. This technique is sufficient for modifying genomic sequence on a scale from one nucleotide to several thousand base pairs. The method may fall short, however, when attempting to alter entire mouse genes that often extend over 10 s or 100 s of thousands of base pairs.

In these instances, other genetic engineering technologies are employed including such methods as random transgenesis^18,19^, targeted transgenesis^20,21^, and recombinase-mediated cassette exchange (RMCE)^22,23^. Each of these methods has its drawbacks as well. For example, random transgenic methods deviate from genome modification at the cognate endogenous locus, sufficing to allow transgenes to integrate randomly (where they are subject to variegated expression). During targeted transgenesis, transgenes may be directed specifically to standardized safe harbor sites to limit this position-effect variegation but even here the transgenes are unlinked to their endogenous cognate genes. Like the related RMCE method, targeted transgenesis may involve the use of antibiotic selection cassettes flanked by recombinase-binding sites. In addition to the added complexity, deleting these selection cassettes requires breeding to specific recombinase-expressing mice thereby prolonging strain development^24–27^.

With the advent of CRISPR technologies many new avenues have opened^28^. For example, by dramatically increasing the frequency of DSBs at specified sites, gene-targeting need no longer be married to the culture of ESCs or the use and removal of selection cassettes^29^. In fact, in mice, most experiments begin with the microinjection of *Cas9* mRNA and single guide RNAs (sgRNAs), (and when needed, donor molecules) into single-cell zygotes^30^. Furthermore, in species where ESC technology is lacking, CRISPR technology is a viable alternative, a fact that has opened gene-editing experimentation to a wide variety of strains and a broad range of species from bacteria to humans^6^. However, here again, DNA modifications have generally been limited to physical extents on the order of a few to a few thousand base pairs. Moreover, the effect of homology arm length on CRISPR-associated HR is only beginning to be elucidated^31^.

One interesting new approach is that of Yoshimi, *et al*.^32^, that sidesteps HR altogether. With this method donor molecules are injected into zygotes along with sgRNAs and single-stranded oligodeoxynucleotides (ssODNs). Donor molecules integrate seamlessly, presumably by a process of ssODN-mediated end joining.

**Figure 1:**
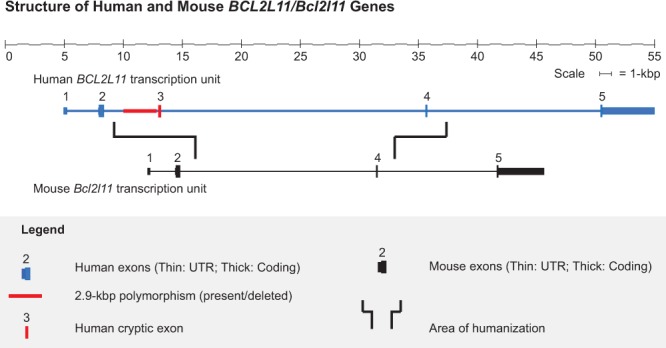
Structure of Human and Mouse *BCL2L11/Bcl2l11* Genes. A simplified schematic of the two gene structures [Mouse *Bcl2l11*, University of California-Santa Cruz (UCSC) transcript isoform 1; human *BCL2L11*, UCSC transcript isoform 1] is shown. Driving the design of this engineering experiment was the desire to humanize the central region of the mouse *Bcl2l11* gene to investigate a cancer-associated 2.9-kbp deletion polymorphism.

**Figure 2:**
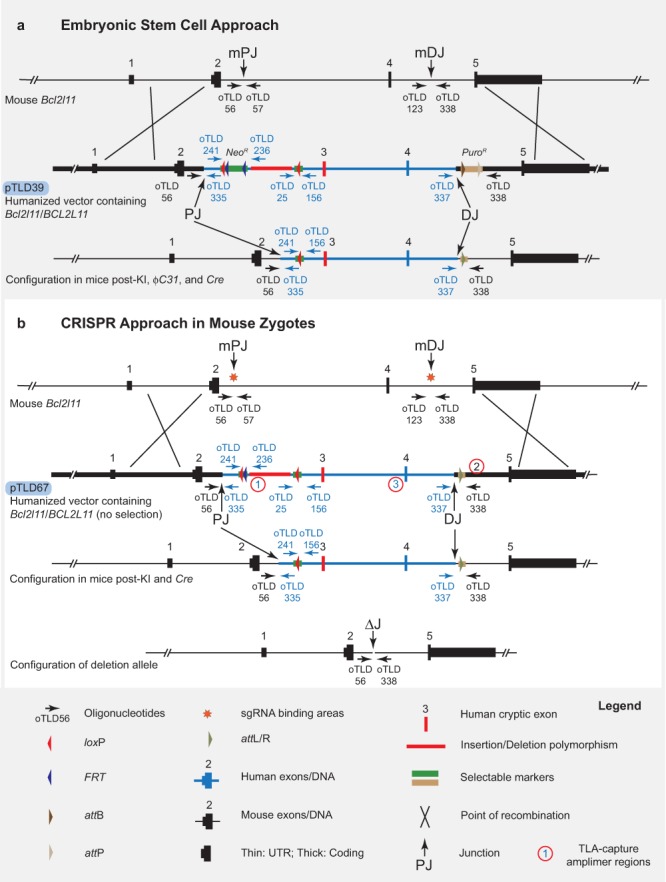
(**a**) ESC/Blastocyst Approach in Mouse Embryonic Stem Cells. The mouse *Bcl2l11* locus, a gene targeting vector (pTLD39), and the modified locus are shown. A gene-targeting vector/donor molecular was constructed placing a 25-kbp segment of the human *BCL2L11* gene between mouse homology arms, placing removable selectable marker cassettes at each end of the human segment, and placing *lox*P sites around a 2.9-kbp segment of human DNA deleted in 12% of the East Asian population (vector names, blue pTLD labels; guide binding areas, orange stars; genotyping oligonucleotide binding sites, oTLD-labelled arrows; proximal junction on mouse locus, mPJ; distal junction on mouse locus, mDJ; proximal mouse/human junction, PJ; distal mouse/human junction, DJ; Targeted Locus Amplification capture amplimers; circled numbers). See text for details. (**b**) CRISPR/Zygote Approach in Mouse Zygotes. The mouse *Bcl2l11* locus, a gene targeting vector (pTLD67), and the modified locus are shown. Vector is as in “a.” above after removal of the *Neo*^*R*^ and *Puro*^*R*^ selection cassettes that are not necessary with the CRISPR/Zygote Approach (vector names, blue pTLD labels; genotyping oligonucleotide binding sites, oTLD-labelled arrows; proximal junction on mouse locus, mPJ; distal junction on mouse locus, mDJ; proximal mouse/human junction, PJ; distal mouse/human junction, DJ; junction over the deletion, ΔJ). See text for details.

In an effort to complement approaches such as those described above, we sought to test the limits of HR-mediated CRISPR knock-in technology, despite the associated technical uncertainties. Specifically, we aimed to test the physical extent to which mouse genomic DNA could be replaced with donor (in this case, human) DNA at an orthologous locus. Driving our efforts was the desire to create a whole animal model that would replace 17 kbp of the mouse *Bcl2l11* gene with the corresponding segment of human *BCL2L11*, including a conditionally removable segment (2.9-kbp) of intron 2, a cryptic human exon immediately 3′ of this, and a native human exon some 20 kbp downstream (Fig. 1)^33^. Using two approaches, we first carried out the replacement by employing a combination of bacterial artificial chromosome (BAC) recombineering, classic ESC targeting, dual selection, and recombinase-driven cassette removal (Fig. 2a; hereafter referred to as our ESC/Blastocyst Approach)^34^. In the second approach, we used the same vector (devoid of its selectable marker cassettes), microinjecting it along with redundant sgRNA guides and *Cas9* mRNA into mouse zygotes (Fig. 2b; hereafter referred to as our CRISPR/Zygote Approach). In both instances, we were able to achieve humanization of *Bcl2l11* to the extent designed, remove all selection cassettes, and demonstrate the functionality of the conditionally removable, *loxP*-flanked, 2.9-kbp intronic segment.

Our latter result represents one of the largest segments of mouse DNA to be replaced by an orthologous human DNA using a CRISPR-directed approach with zygotic injection, to date. This study confirms that a minimum of at least 25 kbp of genomic DNA can be effectively humanized in mouse, and provides a foundation for further technical optimization in mouse and specialization for use in other species.

## Methods

### Husbandry

All mice were obtained from The Jackson Laboratory (JAX; Bar Harbor, ME, USA), housed on a bedding of white pine shavings, and fed NIH-31 5K52 (6% fat) diet and acidified water (pH 2.5 to 3.0), *ad libitum*. All experiments were performed with the approval of The Jackson Laboratory Institutional Animal Care and Use Committee (IACUC) and in compliance with the Guide for the Care and Use of Laboratory Animals (8^th^ edition) and all applicable laws and regulations. The findings presented here have been prepared in close accordance with the ARRIVE Guidelines for reporting research performed *in vivo*^35^.

### Preparation of the targeting vectors/donor molecules

We designed targeting vectors/donor molecules (Fig. 2a,b) with three objectives in mind — 1), to humanize a central segment of the *BCL2L11*/*Bcl2l11* gene; 2), to place selectable markers immediately 5′ and 3′ of the humanized segment; and 3), to flank a 2,903-bp region within one of the humanized introns with *loxP* sites in order to model a disease-associated deletion observed in 12% of the East Asian population^33^.

Specifically, we constructed targeting vectors/donor molecules containing a 27,282-bp central segment of the human *BCL2L11* gene flanked by 12,773- and 26,632-bp homology arms (consisting of the proximal and distal regions of the mouse *Bcl2l11* gene), respectively. These constructs were designed such that they could be used both for homologous recombination in embryonic stem cells (ESCs), as well as for a CRISPR/*Cas9* knock-in approach (Fig. 2a,b).

Additional detail on the construction of targeting vectors/donor molecules is provided in the Supplementary Materials (Supplementary Fig. 1 and Supplementary Table 1).

### Electroporation

For our ESC/Blastocyst Approach, we electroporated 25 µg of linear pTLD39 DNA into 1.5 × 10^7^ cells of the JM8-A3 (Strain: C57BL/6 N) line of mouse embryonic stems cells^36^. ESCs were plated, along with mitotically inactivated mouse embryonic fibroblasts (feeders), in ESC + 2i/LIF medium^37^ under selection with Geneticin^®^ (G418, 200 µg/ml, Gibco, Fisher Thermo Scientific, Waltham, MA, USA) for seven days; or with puromycin (0.75 µg/ml, Sigma-Aldrich, St. Louis, MO, USA; three days on selection, four days off)^37^. Surviving ESC clones were propagated on ESC + 2i/LIF medium, karyotyped, further tested for the presence of the puromycin resistance cassette by PCR (oligonucleotides, Integrated DNA Technologies, Inc., Coralville, IA, USA; AccuStart II PCR SuperMix, Quantabio, Inc., Beverly, MA, USA; Eppendorf Mastercycler ep gradient, Eppendorf AG, Hamburg, DEU), and tentatively assessed for homology arm, human insert, and neomycin resistance cassette count by quantitative PCR. Properly targeted clones were microinjected into 3.5-days *post coitum* (dpc) blastocysts (see below).

### sgRNA design

For our CRISPR/Blastocyst Approach, all single-guide RNAs (sgRNAs) were designed using an algorithm available at http://crispr.mit.edu (August and October 2014; sequence type = unique genomic region; target genome = mouse, mm9)^38^. These sgRNAs, shown in Table 1, were designed along two concepts. In the first, the two highest scoring sgRNAs (with one in each orientation) within a 250-bp region were selected from both the 5′ and 3′ ends of the 17-kbp segment of the mouse *Bcl2l11* segment being replaced. In the second, two internal sgRNAs (with one in each orientation) closest to each end of the replaced segment were selected regardless of their overall score. All sgRNAs were designed so as to not create DSBs within the donor vector. Additional detail on each of the sgRNAs is provided in the Supplementary Materials (Supplementary Fig. 2).

**Table 1:** Single-Guide RNAs.

End	Guide Name	Quality Score	Guide Rank	Guide Sequence	PAM Sequence	Guide Length	Design Parameter	PAM Location	Chromosomal Coordinates (mm10)		Strand
5′	G1	88	1st	5′-dAGTTGTACCAGGCATCACCG-3′	TGG	20	OPTIMAL SCORE	UPSTREAM	Chr 2:	128129677-128129696	minus
5′	G2	76	5th	5′-dAAAATATCCACGGTGATGCC-3′	TGG	20	OPTIMAL SCORE	DOWNSTREAM	Chr 2:	128129667-128129686	plus
3′	G3	89	1st	5′-dTACGTGGAGAAGCACCTTAC-3′	AGG	20	OPTIMAL SCORE	UPSTREAM	Chr 2:	128147456-128147475	minus
3′	G4	81	4th	5′-dTGTAAGGTGCTTCTCCACGT-3′	AGG	20	OPTIMAL SCORE	DOWNSTREAM	Chr 2:	128147455-128147474	plus
5′	G5	75	6th	5′-dTGTGGAAGTGGACGAGTTTG-3′	AGG	20	OPTIMAL POSITION	UPSTREAM	Chr 2:	128129606-128129625	minus
5′	G6	63	10th	5′-dACAACTTTTCCCAGATCAGT-3′	TGG	20	OPTIMAL POSITION	DOWNSTREAM	Chr 2:	128129623-128129642	plus
3′	G7	39	20th	5′-dTTATTTAAATAAATACCAAC-3′	AGG	20	OPTIMAL POSITION	UPSTREAM	Chr 2:	128147642-128147661	minus
3′	G8	54	14th	5′-dAGGGTAGCTGGCTGTCCTGT-3′	TGG	20	OPTIMAL POSITION	DOWNSTREAM	Chr 2:	128147624-128147643	plus

Four guides were designed within 250-bp just internal to each end (5′ and 3′) of the mouse *Bcl2l11* gene segment to be replaced. These include two (one in each orientation) with the top design score (optimal score), and two (one in each orientation) located closest to the outermost ends (optimal position) of the mouse *Bcl2l11* gene segment to be replaced. The guides were designed so as to not cause DSBs within the humanized vector itself.

### sgRNA production

Guides were produced according to the method of Bassett, *et al*.^39^. Briefly, oligonucleotides encoding the T7 polymerase binding site and the sgRNA target sequence as well as an oligonucleotide encoding the remainder of the sgRNA sequence were ordered as 4 nmol Ultramers (Integrated DNA Technologies, Inc., Coralville, IA, USA). Guide templates were amplified with JumpStart *Taq* (Sigma-Aldrich, Corp., St. Louis, MO, USA), purified with a Qiagen PCR Purification Kit (Qiagen, Inc., Germantown, MD USA) and quantified by Nanodrop (ThermoFisher Scientific, Inc., Waltham, MA, USA). Transcription, purification and recovery were via MEGAshortscript and MEGAclear kits (Ambion/ThermoFisher Scientific, Inc., Waltham, MA, USA). Guides were analyzed for quality on the Bioanalyzer (Agilent Technologies, Inc., Santa Clara, CA, USA). Aliquots were frozen at −80′C until use. *Cas9* mRNA (*Streptococcus pyogenes* SF370 mammalian codon-optimized CRISPR associated protein 9 mRNA, 5-methylcytidine, pseudouridine, Product Number L-6125) was purchased from TriLink Biotechnologies, Inc. (San Diego, CA, USA). Each set of sgRNAs (50 ng/µl), *Cas9* mRNA (100 ng/µl), and donor DNA (10, 5, or 1 ng/µl) were assembled in a 25 µl volume with microinjection buffer (1 mM Tris-HCl, pH 7.5; 0.1 mM EDTA) (Table 2). The mixtures were centrifuged at 15,000 rpm for 15 minutes prior to microinjection.

**Table 2:** Microinjection Components and Zygotes Injected for the CRISPR/Zygote Approach.

Experiment	Number	1	2	3	4	5	6^a^	7	8^a^
Guides	OPTIMAL-SCORE GUIDE G1			50 ng/µL	50 ng/µL			50 ng/µL	50 ng/µL
	OPTIMAL-SCORE GUIDE G2			50 ng/µL	50 ng/µL			50 ng/µL	50 ng/µL
	OPTIMAL-POSITION GUIDE G5	50 ng/µL	50 ng/µL			50 ng/µL	50 ng/µL		
	OPTIMAL-POSITION GUIDE G6	50 ng/µL	50 ng/µL			50 ng/µL	50 ng/µL		
	OPTIMAL-SCORE GUIDE G3			50 ng/µL	50 ng/µL			50 ng/µL	50 ng/µL
	OPTIMAL-SCORE GUIDE G4			50 ng/µL	50 ng/µL			50 ng/µL	50 ng/µL
	OPTIMAL-POSITION GUIDE G7	50 ng/µL	50 ng/µL			50 ng/µL	50 ng/µL		
	OPTIMAL-POSITION GUIDE G8	50 ng/µL	50 ng/µL			50 ng/µL	50 ng/µL		
Other Reagents	Cas9 mRNA	100 ng/µL	100 ng/µL	100 ng/µL	100 ng/µL	100 ng/µL	100 ng/µL	100 ng/µL	100 ng/µL
	Circular Donor vector	5 ng/µL	1 ng/µL	5 ng/µL	1 ng/µL	10 ng/µL	5 ng/µL	10 ng/µL	5 ng/µL
	Microinjection Buffer	1×	1×	1×	1×	1×	1×	1×	1×
Zygotes Injected		75	75	81	81	69	72	69	76

Each microinjection included either four optimal-score sgRNAs or four optimal-position sgRNAs (two at the 5′-end and two at the 3′-end of the mouse *Bcl2l11* region to be deleted), one of three targeting vector/donor molecule concentrations (1, 5, or 10 ng/µL), and *Cas9* mRNA. Approximately 75 zygotes were microinjected for each experiment.

^a^Experiments 6 and 8 were replicates of Experiments 1 and 3, respectively.

### ESC/blastocyst approach

#### Microinjection

For our ESC/Blastocyst Approach, properly targeted ESCs (12–15 cells per blastocyst) were microinjected into 3.5-dpc blastocysts (B6(Cg)-*Tyr*^*c*−*2J*^/J, JAX Stock # 000058) and the blastocysts transferred to pseudopregnant host dams (CByB6F1/J; JAX Stock # 100009), by standard techniques^40^. The resulting embryos were allowed to go to term; the pups were delivered naturally and reared by the dams until weaning at four weeks of age.

### CRISPR/zygote approach

For our CRISPR/Zygote Approach, microinjection mixes were prepared as described above (Table 2). Approximately 80 C57BL/6NJ (JAX Stock # 005304) zygotes were microinjected (in one to two technical replicates with each microinjection mix described above), transferred to pseudopregnant females (CByB6F1/J) by standard techniques, and allowed to go to term where they were reared by the dams until weaning at four weeks of age.

#### Zygote collection

C57BL/6J (JAX Stock # 000664) donor female mice (age 3 weeks) were superovulated to maximize embryo yield. Each donor female received five International Units (IU) of Pregnant Mare Serum Gonadotropin (PMSG, ProSpec HOR-272) intraperitoneally (IP), followed 47 hours later by 5 IU of human chorionic gonadotropin (hCG, ProSpec HOR-250), IP. Immediately post-administration of hCG, the female was mated with a single C57BL/6J stud male and was checked 22 hours later for the presence of a copulation plug. Females displaying a copulation plug were euthanized and the oviducts excised and placed into M2 medium. Prior to clutch collection the oviducts were placed in M2 medium containing hyaluronidase (Sigma H3506, 0.3 mg/mL). The oocyte clutch was removed by mechanically lysing the ampulla and the clutch was allowed to incubate in the hyaluronidase-containing M2 medium until the cumulus mass had disintegrated to the point of exposing the oocytes/prospective zygotes. The oocytes/prospective zygotes were then transferred through several washes of fresh M2 medium and then, through the process of visual grading, individual identified zygotes were separated and transferred to microdrops of K-RCVL (COOK K-RVCL) medium that were equilibrated under mineral oil (Sigma M8410) for 24 hours in a COOK MINC benchtop incubator (37°C, 5%CO_2_/5%O_2_/N_2_).

#### Microinjection

Zygotes were removed from culture and placed onto a slide containing 150 µL of fresh M2 medium. Microinjection was conducted on a Zeiss Axio Observer.D1 using Eppendorf NK2 micromanipulators in conjunction with Narashige IM-5A injectors. Standard zygote microinjection procedure was followed with special care made to deposit material (including circular vector) into the pronucleus of the subject zygote. Needles for microinjection were pulled fresh daily using WPI TW100F-4 capillary glass and a Sutter P97 horizontal puller. Injected zygotes were removed from the slide and rinsed through three 30 µL drops of equilibrated K-RCVL before being placed into a final 30 µL microdrop of equilibrated K-RCVL where they were subsequently processed for embryo transfer (via the oviduct) on the day of injection.

#### Transfer

Zygotes processed for same day transfer were removed from culture and placed in a 1.8 mL screw-top tube (Thermo Scientific 363401) containing 900 µL of pre-warmed M2 medium for transport to the surgical station. The zygotes were removed from the tube and placed into culture (K-RCVL under oil, COOK MINC benchtop incubator 37 °C, 5%CO_2_/5%O_2_/N_2_). At the time of transfer the zygotes were removed from culture, placed into pre-warmed M2 medium, and transferred via the oviduct into 0.5 days *post coitum* (dpc) pseudopregnant CByB6F1/J females (age 9–11wks).

### Genotyping

Potential founder (Generation P_0_) chimeric mice, arising from the microinjection of 3.5-dpc blastocysts (ESC/Blastocyst Approach) or 1-cell zygotes (CRISPR/Zygote Approach), and their progeny (Generation N_1_) were genotyped using the oligonucleotides described in Supplementary Table 2. As shown (Fig. 2a,b), these oligonucleotides were used in pairs, in separate PCR reactions designed to amplify DNA across: 1) the proximal (mPJ) and distal (mDJ) junctions, including CAS9 binding sites, of intact (or small INDEL-containing) mouse alleles, 2) the proximal (PJ) and distal (DJ) mouse/human junctions of humanized alleles (or randomly integrating transgenes), and 3) the breakpoints of any deletion-bearing (ΔJ) alleles. Proximal (P2.9), distal (D2.9), and deletion-spanning (Δ2.9) assays were designed around the 2.9-kbp polymorphism, as well. Other less likely forms of variation (larger deletions, inversions, *etc*.), although possible, were not assessed directly by genotyping.

### Sanger sequencing

For more detailed analysis of specific alleles, PCR products from genotyping reactions were purified and sequenced by JAX Scientific Services according to the method developed by Sanger^41^. PCR products were purified using HighPrep PCR magnetic beads (MagBio Genomics, Gaithersburg, MD USA). Cycle sequencing was performed using a BigDye Terminator Cycle Sequencing Kit, version 3.1 (Applied Biosystems, Foster City, CA USA). Sequencing reactions contained 5 µl of purified PCR product (3–20 ng) and 1 µl of oligonucleotide at a concentration of 5 pmol/µl. Sequencing reaction products were purified using HighPrep DTR (MagBio Genomics, Gaithersburg, MD USA). Purified reactions were run on an Applied Biosystems 3730xl DNA Analyzer (Applied Biosystems, Foster City, CA, USA). Sequence data were analyzed using Sequencing Analysis Software, version 5.2 (Applied Biosystems, Foster City, CA, USA). Resulting sequence (.abi) files were imported into Sequencher, version 5.0.1 (Gene Codes Corporation, Ann Arbor, MI, USA), for further analysis.

### Droplet digital PCR

For mice of various genotypes derived from our most promising CRISPR founder, DNA samples were extracted from whole blood using the Qiagen QIAMP DNA mini kit (Qiagen, N.V., Venlo, NED), and quantified using a NanoDrop ND1000 spectrophotometer (ThermoFisher, Inc., Waltham, MA, USA). Copy number of the 5′ homology arm, 3′ homology arm, mouse *Bcl2l11* gene, and human *BCL2L11* gene was determined by Droplet Digital PCR (ddPCR) Copy Number Variation Assays (Bio-Rad Laboratories, Inc., Hercules, CA, USA). Each 22 µl PCR reaction contained 50 ng of DNA template, 10 µl of the ddPCR Supermix for probes (no dUTP), 1 µl of the FAM target oligonucleotides/probe (proprietary, Bio-Rad Laboratories), 1 µl of the HEX reference oligonucleotides/probe (proprietary, Bio-Rad Laboratories), and 10U *Hae*III restriction enzyme (New England BioLabs, Ipswich, MA, USA). Droplets were generated on the QX200 Automated Droplet Generator, using the oil recommended for probes. Plates were heat-sealed with foil, using a PX1 PCR plate sealer set at 180 °C for 5 seconds. A C1000 Touch thermal cycler was used for PCR. Thermocycling conditions consisted of 1 cycle at 95 °C for 10 minutes, 40 cycles of denaturation at 94 °C for 30 seconds, with annealing and extension at 60 °C for 1 minute, and enzyme deactivation at 98 °C for 10 minutes. Droplets were read using a QX200 Droplet Reader. Analysis of the ddPCR data was performed using QuantaSoft software (version1.7.4.0917).

### Genetic mapping

To show that the human segment of *BCL2L11* had replaced its mouse counterpart in the orthologous *Bcl2l11* locus, we used genetic mapping to localize the humanized segment of the *BCL2L11/Bcl2l11* gene. Two backcrosses were established using the following approach. First, FVB/NJ (JAX Stock # 001800) females were crossed to C57BL/6NJ males carrying the humanized segment to obtain first-filial generation (F_1_) *i*.*e*., hybrid (FVB B6N F1/J) progeny. Progeny were then genotyped for the presence of the humanized segment. Males carrying the human sequence (FVB B6N F1/J-*BCL2L11*) were backcrossed to either FVB/NJ females or C57BL/6NJ females to generate second-generation backcross (N_2_) progeny.

These backcross schemes can be written as follows:

C57BL/6NJ X FVB B6N F1/J-*BCL2L11*

FVB/NJ X FVB B6N F1/J-*BCL2L11*

N_2_ progeny from each backcross (along with appropriate controls) were genotyped using KASP-chemistry (KASP chemistry is based on the use allele-specific primers with two distinct fluorophores that are quenched in the unamplified sample. After amplification, relief of quenching results in amplified fluorescence from the alleles present in each sample, be it allele 1, allele 2, or both. Details at http://www.lgcgroup.com; LGC Ltd., Teddington, UK) across a set of approximately 150 single-nucleotide polymorphism (SNP) markers (also available through LGC Ltd.) distributed roughly equally across the mouse genome (those from Chr 2 are further described in Supplementary Table 3). Concordance between each marker in the set and the humanized segment was calculated by chi-square (χ^2^) analysis.

### Targeted locus amplification (TLA)

TLA was performed at Cergentis (Cergentis, B.V., Utrecht, Netherlands) according to the manufacturer’s recommendations. Each of three oligonucleotide sets (Fig. 2b) was used in individual TLA amplifications. PCR products were purified and libraries were prepared using the Illumina NexteraXT protocol (Illumina, Inc., San Diego, CA, USA) and sequenced on an Illumina sequencer.Reads were mapped to the mouse genome (mm 10) using BWA-SW, a Smith-Waterman alignment tool^42^. This allows partial mapping that is optimally suited for identifying break-spanning reads.

### Data and materials availability

The mouse strains described in this work have been cryopreserved, or are maintained, at The Jackson Laboratory and may be accessed by contacting the corresponding author and mentioning JAX Stock (JR) # 27215, # 29083, and # 27561.

Final vector sequences described in this work have been deposited with GenBank and may be accessed under Accession Numbers MG711909 and MG711910. The vectors themselves have not been deposited with Addgene due to restrictions imposed by a Material Transfer Agreement with Children’s Hospital Oakland Research Institute (CHORI; Oakland, CA, USA), the source of mouse and human BACs used in vector construction.

## Results

### ESC/blastocyst approach

**Figure 3:**
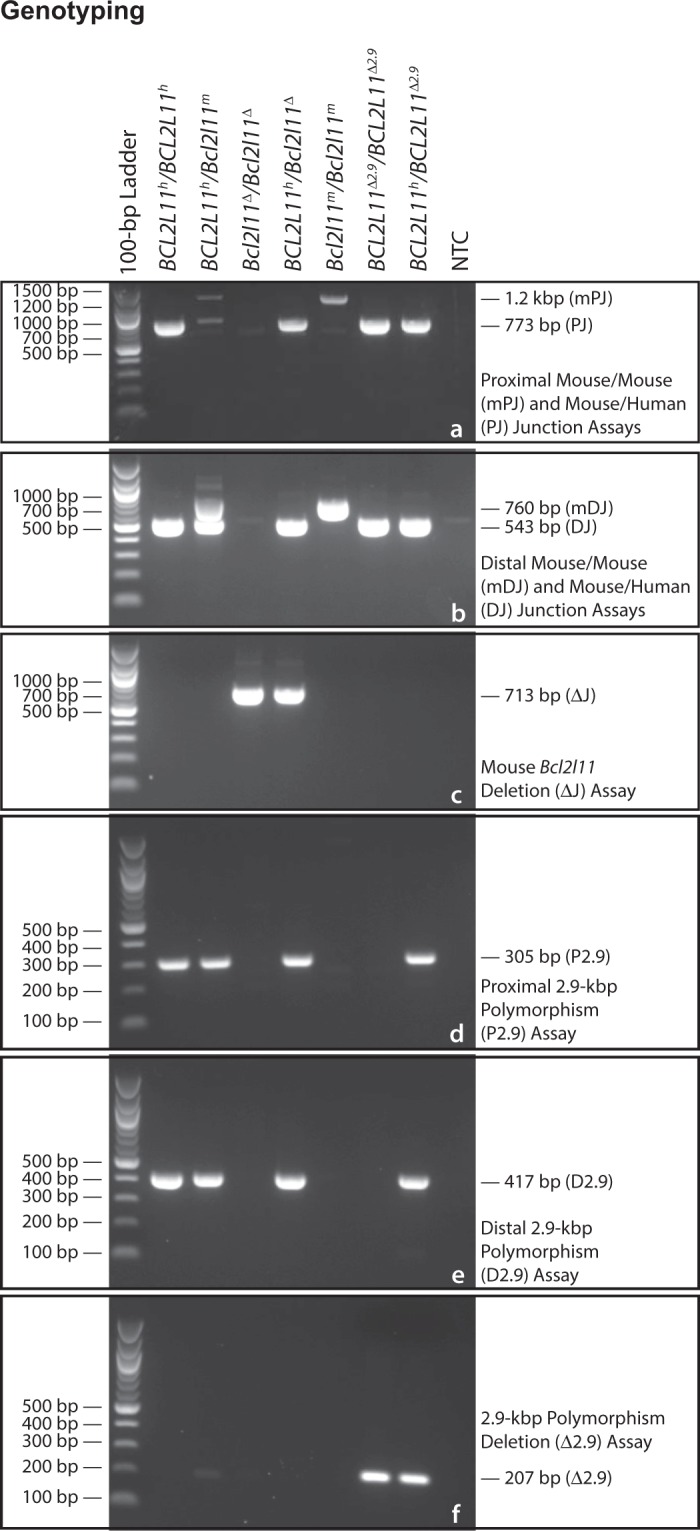
Genotyping. Genotyping Assays for various genotypes of mice from our CRISPR/Zygote Approach are shown. (**a**) Multiplex genotyping with the mPJ and PJ assays identifies the proximal mouse/mouse and mouse/human junctions, respectively. (**b**) Multiplex genotyping with the mDJ and DJ assays identifies the distal mouse/mouse and mouse/human junctions, respectively. (**c**) Genotyping with the ΔJ assay identifies a 17-kbp deletion within the mouse *Bcl2l11* gene. (**d**) Genotyping with the P2.9 assay identifies the 5′ end of the 2.9-kbp polymorphic segment. (**e**) Genotyping with the D2.9 assay identifies the 3′ end of the 2.9-kbp polymorphic segment. (**f**) Genotyping with the Δ2.9 assay identifies *Cre*-*lox*P-mediated deletion of the 2.9-kbp polymorphic segment. *BCL2L11*^*h*^, humanized *BCL2L11* allele; *Bcl2l11*^*m*^, wildtype mouse *Bcl2l11* allele; *Bcl2l11*Δ, mouse *Bcl2l11* deletion allele; *BCL2L11*Δ^*2*.*9*^, humanized *BCL2L11* 2.9-kbp deletion allele. Gel images are cropped.

Following electroporation of the pTLD39 vector into the JM8-A3 line of ESCs and selection on G418, we assayed 89 surviving clones for the presence of the puromycin resistance cassette by PCR. Of these, twenty-seven contained the puromycin cassette and were subjected to puromycin selection. Surprisingly, of these, only four clones survived to be assessed by quantitative PCR for homology arm, insert, and neomycin resistance cassette count (data not shown). One clone passed all of these tests for proper targeting of the central human *BCL2L11* segment to the endogenous mouse *Bcl2l11* gene. ESCs from this clone were microinjected into blastocysts resulting in nine high-quality (>50% chimerism as assessed by coat color) chimeras. The four highest quality male chimeras were mated to C57BL/6NJ females resulting in two independent instances of germline transmission of the humanized allele. Although presumably identical, independent lines (genetic background: C57BL/6JN) were developed from each instance. Mating males with B6N.Cg-Tg(*Sox2-Cre*)1Amc/J (JAX Stock # 014094) female mice resulted in progeny in which the *loxP*-flanked 2.9-kbp human intronic segment was deleted, as designed (Fig. 3).

### CRISPR/Zygote approach

In each of our CRISPR/Zygote Approach experiments, we employed four sgRNAs, two directed near the proximal end of the region of the mouse *Bcl2l11* gene to be replaced and two directed near the distal end (Supplementary Fig. 2). In half of the experimental conditions that we tested, these four guides were those with the highest guide scores. In the remaining half of the experimental conditions that we tested, these four guides were those with the most terminal positions within the region of the mouse *Bcl2l11* gene to be replaced (Table 2). We hypothesized that the redundancy of sgRNAs used in each of our experiments might be optimizing for (either or both of) the following reasons: first, that if one or the other sgRNA from a pair were inefficient at creating DSBs, the other might perform the task; and second, that if the creation and repair of DSBs were in a dynamic equilibrium, the presence of two closely spaced sgRNAs might cause DSBs to persist for a longer duration allowing more time for HR to occur. The results of these experiments are described here.

At term, a total of 89 pups were born of which five were stillborn and three did not survive to four weeks of age. The remaining eighty-one mice were weaned and are categorized among experiments as shown (Table 3).

**Table 3:** CRISPR/Zygote Approach Microinjection Results.

Experiment Number	Donor Vector (ng/µL)	Guide Set	Zygotes Injected/Transferred	Newborns		Liveborn^a^		Stillborn^b^		Lost Before Wean^c^		Weaned^d^		Founders^e^	
1 and 6 combined	5	OPTIMAL POSITION (G5-G8)	147	33	22.4%	30	90.9%	3	9.1%	1	3.3%%	29	96.7%	1^f^	3.4%
2	1	OPTIMAL POSITION (G5-G8)	75	8	10.7%	8	100.0%	0	0.0%	0	0.0%	8	100.0%	1^g^	12.5%
4	1	OPTIMAL SCORE (G1-G4)	81	17	21.0%	17	100.0%	0	0.0%	1	5.9%	16	94.1%	0	0.0%
7	10	OPTIMAL SCORE (G1-G4)	69	8	11.6%	7	87.5%	1	12.5%	0	0.0%	7	100.0%	1^g^	14.3%
8	5	OPTIMAL SCORE (G1-G4)	76	23	30.3%	22	95.7%	1	4.3%	1	4.5%	21	95.5%	0	0.0%
		**Totals**	448	89	19.9%	84	94.4%	5	5.6%	3	3.6%	81	96.4%	3	3.7%

Mice recovered from the CRISPR/Zygote Approach microinjection experiments (described in Table 2) are shown. These experiments tested permutations of two design parameters — targeting vector/donor DNA concentration (at 1, or 5, or 10 ng/µL) and sgRNA design (optimal score or optimal position). Experiments 1 and 6 are replicates; the results of these experiments are combined. Experiments 3 and 5 were technical failures resulting in no wean-age mice (see Supplementary Table 4 for details). Experiments 2, 6, and 7 generated single founder animals. See Table 4 for details.

^a^Percentage calculated as Liveborn/Newborns × 100.

^b^Percentage calculated as Stillborn/Newborns × 100.

^c^Percentage calculated as Lost Before Wean/Liveborn × 100.

^d^Percentage calculated as Weaned/Liveborn × 100.

^e^Percentage calculated as Founders/Weaned × 100.

^f^Proper integration and transmission confirmed. See Tables 4, 5, and text for details.

^g^Was not transmitted through the germline. See Tables 4, 5, and text for details.

Both Experiment 3 (highest scoring guides, 5 ng/µL donor DNA) and Experiment 5 (guides closest to ends, 10 ng/µL donor DNA) (Table 2) resulted in no viable pups remaining at wean-age (Supplementary Table 4). Despite these results Experiment 7 (conducted with a donor DNA concentration equal to that of Experiment 5, *i*.*e*., 10 ng/µL) and Experiment 8 (a replicate of Experiment 3) resulted in seven and 21 pups (at weaning), respectively, suggesting that the lack of pups in Experiments 3 and 5 was due to technical failure rather than anything systematically wrong with the experimental design.

To genotype these 81 progeny, PCR assays were designed to span each of the following regions: the proximal breakpoint of the mouse *Bcl2l11* gene (PCR Assay mPJ), the distal breakpoint of the mouse *Bcl2l11* gene (PCR Assay mDJ), the proximal mouse *Bcl2l11*/human *BCL2L11* junction (PCR Assay PJ), the distal mouse *Bcl2l11*/human *BCL2L11* junction (PCR Assay DJ), and the 17-kbp mouse region (to be replaced) were it deleted (PCR Assay ΔJ) (see Figs 2a,b, 3a–c, and Supplementary Table 2). The results of these experiments are shown (Table 4).

**Table 4:** Detection of Small Insertion/Deletion (INDEL), Vector Integration, and Larger Deletion Events Among CRISPR/Zygote Approach Founder (P_0_) Mice.

Experiment Number	Injected DNA Concentration	PCR Assay →	mPJ		mDJ		PJ	DJ	ΔJ
		Oligonucleotides →	oTLD56 (forward) and oTLD57 (reverse)		oTLD123 (forward) and oTLD338 (reverse)		oTLD56 (forward) and oTLD335 (reverse)	oTLD337 (forward) and oTLD338 (reverse)	oTLD56 (forward) and oTLD338 (reverse)
		↓ Founders/sgRNAs →	Optimal-Position Guides						
			G5	G6	G8	G7			
Experiment 1	5 ng/µL	9	−	−	−	−	−	−	−
		1	−	−	5-bp deletion	−	−	−	−
		1	−	−	1-bp deletion	−	−	−	−
		1	6-bp deletion	5-bp deletion	1-bp deletion	−	−	−	−
Experiment 2	1 ng/µL	5	−	−	−	−	−	−	−
		1	−	−	−	−	+	+	−
		1	6-bp deletion	9-bp deletion	1-bp deletion	−	−	−	−
		1	−	−	1-bp deletion	−	−	−	−
Experiment 6	5 ng/µL	12	−	−	−	−	−	−	−
		1	6-bp deletion	5-bp deletion	−	−	−	−	−
		1	−	−	5-bp deletion	−	−	−	−
		1	−	double peaks	−	−	+	+	+
		1	−	−	5-bp deletion	−	−	−	−
		1	6-bp deletion	5-bp deletion	−	−	−	−	−
Totals	Mutational Events		**4**	**5**	**7**	**0**	**2**	**2**	**1**
	Founders Tested		**37**	**37**	**37**	**37**	**37**	**37**	**37**
	Percentage		**10.8%**	**13.5%**	**18.9%**	**0.0%**	**5.4%**	**5.4%**	**2.7%**
**Experiment** **Number**	**Injected** **DNA** **Concentration**	**PCR Assay →**	**mPJ**		**mDJ**		**PJ**	**DJ**	**ΔJ**
		**Oligonucleotides →**	**oTLD56 (forward) and oTLD57 (reverse)**		**oTLD123 (forward) and oTLD338 (reverse)**		**oTLD56 (forward) and oTLD335 (reverse)**	**oTLD337 (forward) and oTLD338 (reverse)**	**oTLD56 (forward) and oTLD338 (reverse)**
		**↓ Founders/sgRNAs →**	**Optimal-Position Guides**						
			**G2**	**G1**	**G3**	**G4**			
Experiment 4	1 ng/µL	15	−	−	−	−	−	−	−
		1	−	−	−	39-bp deletion	−	−	−
Experiment 7	10 ng/µL	6	−	−	−	−	−	−	−
		1	−	2-bp deletion	−	−	+	+	+
Experiment 8	5 ng/µL	19	−	−	−	−	−	−	−
		1	−	−	−	1-bp deletion	−	−	−
		1	−	−	−	14-bp deletion	−	−	−
Totals	Mutational Events		**0**	**1**	**0**	**3**	**1**	**1**	**1**
	Founders Tested		**44**	**44**	**44**	**44**	**44**	**44**	**44**
	Percentage		**0.0%**	**2.3%**	**0.0%**	**6.8%**	**2.3%**	**2.3%**	**2.3%**

Reaction products from the PCR assays mPJ and mDJ were sequenced and analyzed to identify the nature and frequency of INDELs induced within potential founder (P_0_) mice of Experiments 1, 2, 4, 6, 7, and 8 by sgRNAs (guides) G1 through G8, as shown. INDEL frequencies ranged from 0.0% (G2, G3, and G7) to 18.9% (G8). Reaction products from PCR assays PJ, DJ, and ΔJ were sequenced and analyzed to identify incorporation of the *BCL2L11* humanization vector (PJ and DJ) or deletion across the entirety of the mouse *Bcl2l11* region being replaced (ΔJ). In Experiment 2, the P_0_ mouse (male) tested positive for both the proximal (PJ) and distal (DJ) mouse/human junctions by PCR. In Experiment 6, the P_0_ mouse (female) tested PCR-positive for both the proximal (PJ) and distal (DJ) mouse/human junctions and the deletion-bearing allele (ΔJ). In Experiment 7, the P_0_ mouse (male) also tested PCR-positive for both the proximal (PJ) and distal (DJ) mouse/human junctions and the deletion-bearing allele (ΔJ). Because P_0_ animals may be mosaic, each founder was bred in an attempt to establish germline transmission of each modified allele. See Table 5 and text for details.

**Figure 4:**
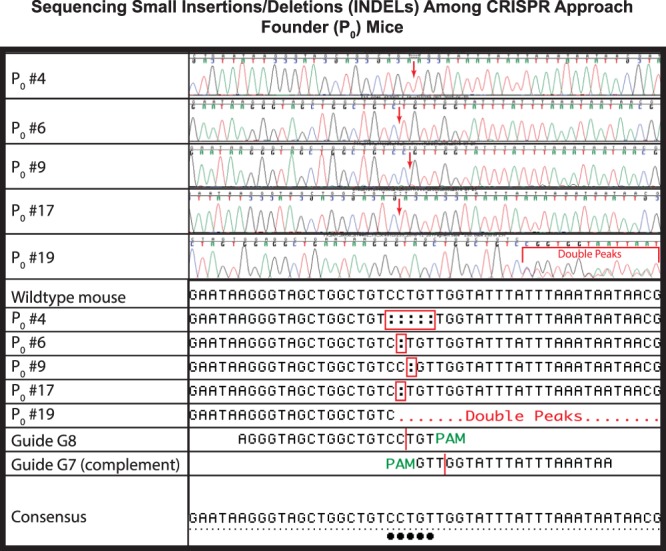
Sequencing Small Insertions/Deletions (INDELs) Among CRISPR/Zygote Approach Founder (P_0_) Mice. Sanger sequencing chromatograms and deduced sequences are shown for five potential founder (P_0_) mice (P_0_ #s 4, 6, 9, 17, and 19) from the CRISPR/Zygote Approach. Red arrows, location of INDELs within chromatograms; red bracket, location of double peaks within chromatograms; red rectangles, location of INDELs within deduced sequence; red dots, location of double peaks within deduced sequence; vertical red lines, point of DSB creation by Guides G7 and G8; green PAM, location of NGG PAM sites; black dots, deviations from wildtype sequence which is also shown.

PCR assays mPJ and mDJ amplify the proximal and distal regions of the mouse *Bcl2l11* gene, respectively, where the sgRNA pairs were designed to act. We sequenced these amplification products (Sanger method) to identify potential small insertion/deletion (INDEL) events. Although under all experimental conditions, apparently unmodified DNA was most commonly observed (range 81.1% to 100.0%), INDELs were observed for five of eight sgRNAs (2.3% to 18.9%). In many instances, sequenced products provided well-organized traces suggestive of mono-allelic amplification, the second allele presumably failing to amplify due to an underlying INDEL which had deleted an oligonucleotide binding site (Fig. 4, P_0_ animals # 4, 6, 9, and 17). In addition, in some instances, sequencing traces were consistent with the amplification of at least two alleles (Fig. 4, P_0_ animal # 19).

As further noted in Table 4, PCR assays designed to span each of the proximal (PJ) and distal (DJ) mouse/human junctions identified three founders that were positive for both (Experiment 2, guides closest to ends, 1 ng/µL donor DNA; Experiment 6, guides closest to ends, 5 ng/µL donor DNA; and Experiment 7, highest scoring guides, 10 ng/µL donor DNA). PCR assays designed to span the 17-kbp mouse region (ΔJ) to be replaced (were it deleted) identified two of the three founders described above (Experiment 6, guides closest to ends, 5 ng/µL donor DNA; and Experiment 7, highest scoring guides, 10 ng/µL donor DNA).

To further explore the inheritance of these genetic changes, we mated the founder mice (Generation P_0_) from Experiments 2 (PJ^+^ DJ^+^ ΔJ^−^), 6 (PJ^+^ DJ^+^ ΔJ^+^), and 7 (PJ^+^ DJ^+^ ΔJ^+^) to C57BL/6J mice and genotyped their progeny. The results of these analyses are shown in Table 5. As shown, the P_0_ mouse (male) from Experiment 2 (guides closest to ends, 1 ng/µL donor DNA), although PJ^+^ and DJ^+^, failed to transmit the humanized allele to any of 29 of its first-generation backcross (N_1_) progeny suggesting that the P_0_ mouse is mosaic with a germline consisting primarily of cells that do not harbor the humanization construct or anticipated deletion.

**Table 5:** Germline Transmission of CRISPR-Modified Alleles.

Founder from Experiment Number	Founder Genotype	PCR Assay →	PJ	DJ	ΔJ	Percentage	GLT of Humanized Construct by Founder?	GLT of Mouse *Bcl2l11* Deletion by Founder?
		Oligonucleotides →	oTLD56 (forward) and oTLD335 (reverse)	oTLD337 (forward) and oTLD338 (reverse)	oTLD56 (forward) and oTLD338 (reverse)			
		↓ N_1_ Progeny Classes						
2	PJ^+^ DJ^+^ ΔJ^−^	50	−	−	−	100.0%	No	No
6	PJ^+^ DJ^+^ ΔJ^+^	17	−	−	**+**	54.8%	Yes	Yes
		14	**+**	**+**	−	45.2%		
7	PJ^+^ DJ^+^ ΔJ^+^	42	−	−	−	75.0%	No	Yes
		14	−	−	**+**	25.0%		

Genotyping results for progeny mice recovered from the breeding of CRISPR-modified allele founders are shown. In Experiment 2, the founder mouse (male), although PCR-positive for both the proximal (PJ) and distal (DJ) mouse/human junctions, failed to transmit the modified allele to its progeny. In Experiment 7, the founder mouse (male), although PCR-positive for both the proximal (PJ) and distal (DJ) mouse/human junctions and the deletion-bearing allele (ΔJ), transmitted only the deletion-bearing allele to its progeny. In Experiment 6, the founder mouse (female), testing PCR-positive for both the proximal (PJ) and distal (DJ) mouse/human junctions and the deletion-bearing allele (ΔJ), transmitted single modified alleles to each of its progeny, *i*.*e*., the founder either transmitted the deleted allele or the humanized allele to its progeny, but never both alleles, or a wildtype allele. This result is consistent with the founder from Experiment 6 being a true heterozygote with a genotype of PJ^+^ DJ^+^/ΔJ^+^. See main text for details.

In contrast, the PJ^+^ DJ^+^ ΔJ^+^ P_0_ mouse (male) from Experiment 7 (highest scoring guides, 10 ng/µL donor DNA) transmitted its deletion-bearing allele (ΔJ^+^) to four of its 21 N_1_ progeny. This P_0_ mouse, however, did not transmit the human insertion-bearing allele (PJ^+^, DJ^+^) to any of these 21 mice again suggesting that the P_0_ mouse is mosaic with a germline consisting of relatively few human insertion-bearing cells.

Interestingly, the PJ^+^ DJ^+^ ΔJ^+^ P_0_ mouse (female) from Experiment 6 (guides closest to ends, 5 ng/µL donor DNA) transmitted either a human insertion-bearing allele PJ^+^ DJ^+^ ΔJ^−^ or a deletion-bearing allele PJ^−^ DJ^−^ ΔJ^+^ to all of its 13 N_1_ progeny, but never both, implying that this animal is breeding as a true heterozygote with a genotype of both human insertion- and deletion-bearing alleles (PJ^+^ DJ^+^/ΔJ^+^) at the *Bcl2l11* locus. Subsequent breeding of three select N_1_ mice, two bearing the human insertion (PJ^+^ DJ^+^ ΔJ^−^) and one bearing the deletion (PJ^−^ DJ^−^ ΔJ^+^), gave results consistent with Mendelian expectations. Mating males with B6N.Cg-Tg(*Sox2-Cre*)1Amc/J female mice resulted in progeny in which the *loxP*-flanked 2.9-kbp human intronic segment (Fig. 3d,e) was deleted (Fig. 3f), as designed.

### Droplet digital PCR

**Figure 5:**
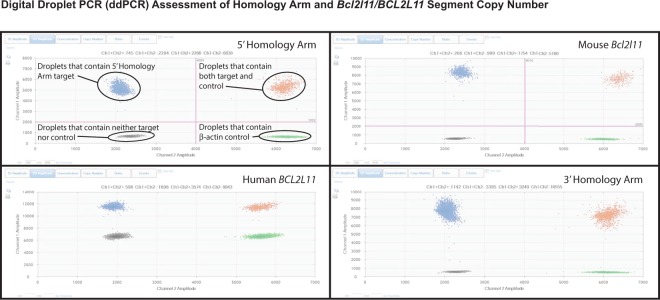
Digital Droplet PCR (ddPCR) Assessment of Homology Arm and *Bcl2l11/BCL2L11* Segment Copy Number. Two dimensional amplitude plots for assessing copy number of the 5′ homology arm, mouse *Bcl2l11* gene, human *BCL2L11* gene, and 3′ homology arm by ddPCR are shown. For any analyzed DNA with a genotype of interest, the fluorescence amplitude within droplets, and the relative number of droplets can be used to determine the relative copy number of two DNA segments, in this case, elements of the *BCL2L11* humanization and a β-actin control. See Table 6 and text for more detail.

To determine the copy number of various DNA segments associated with the integration and deletion events arising from the Experiment 6 founder, we raised mice with various preliminary genotypes and assessed the copy number of the 5′ homology arm, the 3′ homology arm, the mouse *Bcl2l11* gene, and the human *BCL2L11* gene by droplet digital PCR (ddPCR) (Fig. 5). For these experiments mouse genomic DNAs with the following preliminary genotypes were employed: a mouse *Bcl2l11* deletion allele homozygote (Bcl2l11Δ/Bcl2l11Δ), a humanized *BCL2L11* allele/mouse *Bcl2l11* deletion allele heterozygote (*BCL2L11*^*h*^*/Bcl2l11*Δ), a humanized *BCL2L11* allele homozygote (*BCL2L11*^*h*^*/BCL2L11*^*h*^, N = 2), a humanized *BCL2L11* allele/wildtype mouse *Bcl2l11* allele heterozygote (*BCL2L11*^*h*^*/Bcl2l11*^*m*^, N = 2), and a mouse wildtype *Bcl2l11* allele homozygote (*Bcl2l11*^*m*^*/Bcl2l11*^*m*^).

In all instances, copy number of the 5′ homology arm, 3′ homology arm, mouse *Bcl2l11* gene, and human *BCL2L11* gene was as expected based on each animal’s preliminary genotype (Table 6).

**Table 6:** Digital Droplet PCR (ddPCR) Quantification of the 5′ Homology Arm, 3′ Homology Arm, Human *BCL2L11*, and Mouse *Bcl2l11* Segments of C57BL/6 J Mice Humanized by the CRISPR/Zygote Approach.

Genotype Tested	Trial Number	Observed							Expected
		β-actin	5′ Homology Arm	Mouse *Bcl2l11*	Human *BCL2L11*	3′ Homology Arm	Ratio	Copy Number	Copy Number
		Copies/µL	Copies/µL	Copies/µL	Copies/µL	Copies/µL	Relative to β-actin	Diploidy = 2	Diploidy = 2
*Bcl2l11*Δ*/Bcl2l11*Δ	Trial 1	172	170	—	—	—	0.9884	1.98	2
		142	—	0	—	—	0.0000	0.00	0
		162	—	—	0	—	0.0000	0.00	0
		158	—	—	—	158	1.0000	2.00	2
*BCL2L11*^*h*^*/Bcl2l11*Δ	Trial 1	582	596	—	—	—	1.0241	2.05	2
		572	—	0	—	—	0.0000	0.00	0
		979	—	—	510	—	0.5209	1.04	1
		663	—	—	—	684	1.0317	2.06	2
*BCL2L11* ^*h*^ */BCL2L11* ^*h*^	Trial 1	466	465	—	—	—	0.9979	2.00	2
		393	—	0	—	—	0.0000	0.00	0
		400	—	—	407	—	1.0175	2.04	2
		462	—	—	—	475	1.0281	2.06	2
	Trial 2	379	375	—	—	—	0.9894	1.98	2
		390	—	0	—	—	0.0000	0.00	0
		406	—	—	403	—	0.9926	1.99	2
		415	—	—	—	416	1.0024	2.00	2
*BCL2L11* ^*h*^ */Bcl2l11* ^*m*^	Trial 1	336	338	—	—	—	1.0060	2.01	2
		341	—	186	—	—	0.5455	1.09	1
		360	—	—	184	—	0.5111	1.02	1
		322	—	—	—	334	1.0373	2.07	2
	Trial 2	325	343	—	—	—	1.0554	2.11	2
		376	—	189	—	—	0.5027	1.01	1
		306	—	—	143	—	0.4673	0.93	1
		356	—	—	—	344	0.9663	1.93	2
*Bcl2l11* ^*m*^ */Bcl2l11* ^*m*^	Trial 1	326	348	—	—	—	1.0675	2.13	2
		359	—	361	—	—	1.0056	2.01	2
		382	—	—	0	—	0.0000	0.00	0
		343	—	—	—	347	1.0117	2.02	2
NTC	Trial 1	0	0	—	—	—	N/A	N/A	N/A
		0	—	0	—	—	N/A	N/A	N/A
		0	—	—	0	—	N/A	N/A	N/A
		0	—	—	—	0	N/A	N/A	N/A

Mice of various genotypes were assessed by ddPCR for the copy number of various vector components. In all cases, copy number is consistent with proper integration of the humanization vector by HR, as designed.

### Genetic mapping

**Figure 6:**
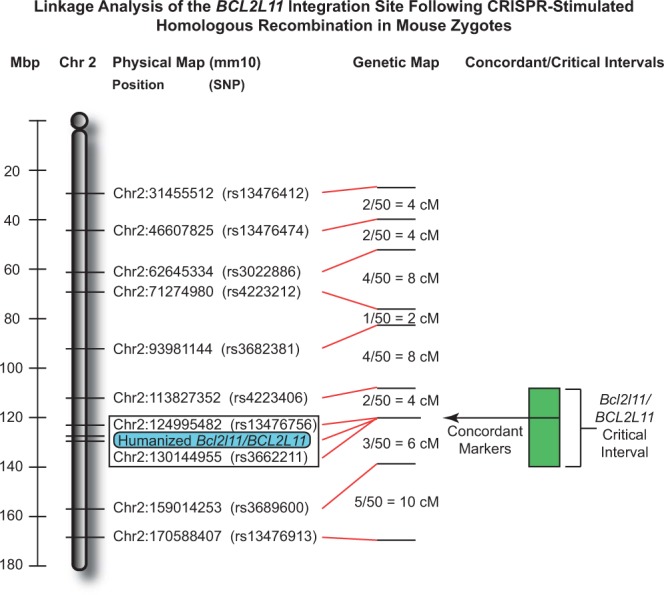
Linkage analysis of the *BCL2L11* integration site following CRISPR-stimulated homologous recombination in mouse zygotes. Shown is the linkage analysis for 22 F_2_ progeny of a C57BL/6NJ X FVBB6NF1/J-*BCL2L11* backcross and 28 F_2_ progeny of an FVB/NJ X FVBB6NF1/J-*BCL2L11* backcross. Linkage and haplotype analyses indicate that the *BCL2L11* vector’s integration has occurred between markers rs4223406 and rs3689600 and its segregation is fully concordant with markers rs13476756 and rs3662211. This result is entirely consistent with integration of the human *BCL2L11* segment within the endogenous mouse *Bcl2l11* gene as designed. Megabasepair positions along Mouse Chromosome 2, the position of mapping SNPs, and genetic distances are shown. Concordant markers are enclosed in a rectangle. The extent of the *Bcl2l11/BCL2L11* critical interval (within which the humanization vector integration is mapped) is shown in green.

We used an outcross-backcross genetic mapping strategy as a means of localizing the insertion site of BAC-derived human *BCL2L11* sequences. Twenty-two N_2_ progeny were analyzed from the C57BL/6NJ X (FVB/NJ X C57BL/6NJ) backcross and twenty-eight progeny from the FVB/NJ X (FVB/NJ X C57BL/6NJ) backcross. Analysis of the data demonstrates strong linkage between the human *BCL2L11* segment and several genetic markers on mouse Chromosome 2 (Fig. 6 and Supplementary Table 3). In the backcross to C57BL/6NJ, the marker with strongest linkage, marker rs13476756, had a log-odds ratio (LOD) of 6.58 (p < 0.004). In the backcross to FVB/NJ, marker rs13476756 had a LOD score of 7.64 (p < 0.0004).

Analysis of individual haplotypes (specifically, points of recombination in samples 261, 263, 266, 303, and 319) further narrows the insertion-critical region to a 45.2-Mbp region from marker rs4223406 (nucleotide 113,827,352) to marker rs3689600 (nucleotide 159,014,253) on Mouse Chromosome 2 (GRC38/mm10), which is consistent with integration into the 36,510-bp mouse *Bcl2l11* gene that spans from nucleotide 128,126,038 to nucleotide 128,162,547. Put another way, this analysis shows that both the mouse *Bcl2l11* gene and the engineered human sequences must be colocalized within a region comprising less than 2% of the mouse genome. We conclude that integration of the human sequence has not occurred randomly, but has indeed occurred by homologous recombination as designed.

### Targeted locus amplification

Due to the large size of the homology arms (>15-kbp) used in our experiment, typical approaches used to confirm proper targeting (Southern blot analysis and long-range PCR) were untenable. Thus, to further address the issue of proper genomic targeting and the precise nature of the integration site, we turned to a more modern technique, targeted locus amplification (TLA)^43^.

TLA is a DNA crosslinking-based technique used to amplify tens of thousands of base-pairs in, and immediately surrounding, a locus of interest. When coupled with next-generation sequencing methods an entire locus of interest can be assessed at base-pair resolution.

We performed TLA with three amplicons (Fig. 2b and Supplementary Table 5) in the *Bcl2l11/BCL2L11* targeted region — the first, at the 5′ end of the human *BCL2L11* segment; the second, at the 3′ end of the human *BCL2L11* segment; and third, in the 3′ (mouse) homology arm.

**Figure 7:**
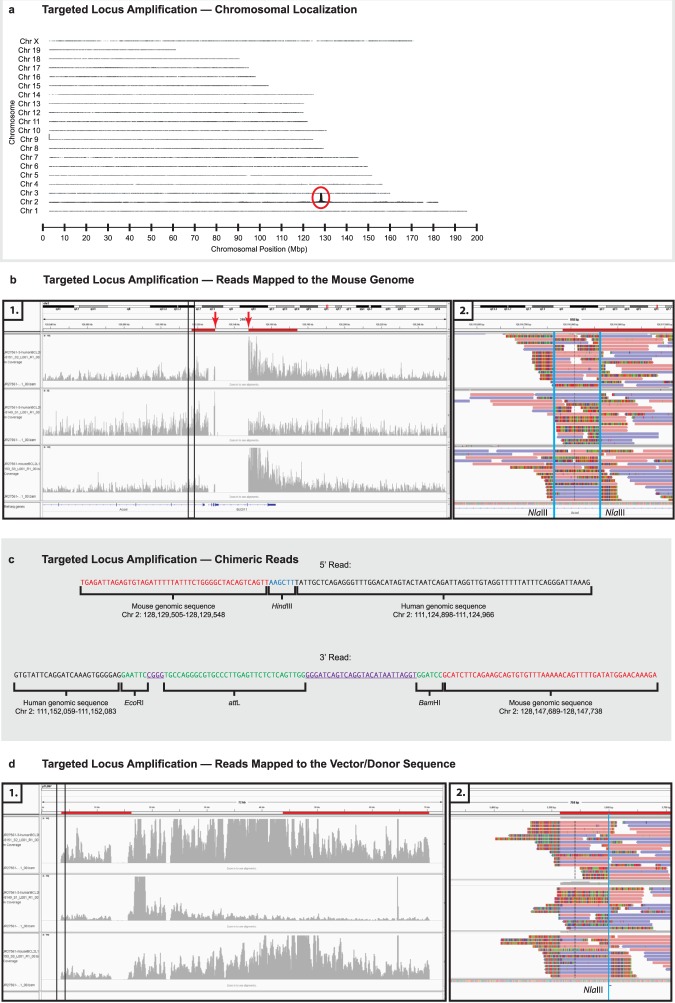
(**a**) Targeted Locus Amplification — Chromosomal Amplification. High-throughput sequencing reads, obtained through the use of the TLA technique with human and mouse *BCL2L11-/Bcl2l11-*derived target amplimers, identify distal Chr2 as the integration site of the humanizing gene targeting vector/donor molecule. This result is entirely consistent with integration of the human *BCL2L11* segment within the endogenous mouse *Bcl2l11* gene as designed (vertical axis, read-depth along each of the mouse chromosomes; horizontal axis, chromosomal position). (**b**) Targeted Locus Amplification — Reads Mapped to the Mouse Genome. 1. High-throughput sequencing reads, obtained through the use of the TLA technique with human and mouse *BCL2L11-/Bcl2l11-*derived target amplimers, localize to the mouse *Bcl2l11* locus outside of the humanized region. This result is entirely consistent with integration of the human *BCL2L11* segment within the endogenous mouse *Bcl2l11* gene as designed (vertical axis, read-depth along the mouse *Bcl2l11* locus for each of three target amplimers; horizontal axis, chromosomal position; red lines, regions of the two mouse homology arms; red arrows, internal boundary of homology arms and location of chimeric human/mouse *BCL2L11/Bcl2l11* sequencing reads; central blue gene structure, *Bcl2l11*; left blue gene structure, an adjacent gene; narrow vertical rectangle, region examined in panel 2). 2. Sequencing reads mapping to the 5′ end of the 5′ homology arm/flanking mouse genome boundary. Sequencing reads spanning the homology arm/genome boundary are contiguous (horizontal pink and purple bands). Fusion reads (horizontal bands with multicolored segments) arise only from nearby *Nla*III sites (*Nla*III-labelled vertical blue lines) and are an artifact of the TLA technology. No fusion reads suggest integration at an ectopic locus. Analysis of the 3′ end of the 3′ homology arm was similar (not shown). (**c**) Targeted Locus Amplification — Chimeric Reads. Representative reads from the 5′ and 3′ mouse/human breakpoints (junctions) of the humanized *BCL2L11/Bcl2l11* locus (red lettering, mouse-derived sequence; black lettering, human-derived sequence; blue lettering, vector-derived *Hind*III site at the 5′ mouse/human junction; green lettering, vector-derived *Eco*RI, *att*L, and *Bam*HI sites at the 3′ mouse/human junction; underlined purple lettering, additional vector-derived sequences). (**d**) Targeted Locus Amplification — Reads Mapped to the Vector/Donor Sequence. 1. High-throughput sequencing reads, obtained through the use of the TLA technique with human and mouse *BCL2L11-/Bcl2l11-*derived target amplimers, localize across the pTLD67 gene targeting vector/donor molecule including mouse and human elements. This result is entirely consistent with integration of the human *BCL2L11* segment within the endogenous mouse *Bcl2l11* gene as designed (vertical axis, read-depth along the pTLD67 vector for each of three target amplimers; horizontal axis, position within the vector; red lines, regions of the two mouse homology arms; narrow vertical rectangle, region examined in panel 2). 2. Sequencing reads mapping to the 5′ end of the 5′ homology arm/vector boundary. Reads arising from the point of integration (horizontal bands with multicolored segments) appear as fusion reads at the homology arm/vector boundary. Additional fusion reads arise only from a nearby *Nla*III site (*Nla*III-labelled vertical blue line) and are an artifact of the TLA technology. No fusion reads suggest continuity into the vector’s backbone. Analysis of the 3′ end of the 3′ homology arm was similar (not shown).

Recovered DNAs were sequenced and mapped to the mouse genome (mm10). As shown (Fig. 7a) the highest coverage of TLA reads, recovered using the 3′ human amplicon, is observed between 125 and 130 Mbp on Chr 2, indicating that the *BCL2L11* containing targeting construct had integrated, as expected (*i*.*e*., within the general region of the mouse *Bcl2l11* gene), and that there had been no off-target integration events in this line of mice. Similar results were obtained with the other two amplicons (data not shown).

More specifically, when coverage around the *Bcl2l11* locus is analyzed at higher resolution, it can be seen that for each of the three amplicons individual TLA-recovered sequences localize to regions of the 5′ and 3′ mouse homology arms (Fig. 7b1) and, most important, some extend in a seamless fashion more laterally into areas of the genome immediately flanking the mouse homology arms, as expected (Fig. 7b2), but nowhere else.

No recovered sequences localize to the central region of the mouse *Bcl2l11* gene, suggesting that the central region has been deleted (*i*.*e*., replaced) by the orthologous integrating human sequence. In fact, at the 5′ and 3′ extremes of the central region of *Bcl2l11* to be replaced, local sequencing reads are chimeric, containing sequences from both the mouse *Bcl2l11* and human *BCL2L11* genes adjoining as originally designed in our targeting vector (Fig. 7c).

Moreover, when aligned to the sequence of our targeting vector itself, TLA-recovered sequences from each of the three amplicons localize across the construct, aligning with the 5′ mouse homology arm, the central region of the human *BCL2L11* gene, and the 3′ homology arm, as designed (Fig. 7d1, d2).

Other evidence supporting presence of the designed integration event includes the absence of TLA-recovered sequence reads corresponding to the vector backbone, the absence of sequence reads indicating unexpected transgene/transgene fusions, and the absence of sequence reads indicating unexpected transgene/genome fusions.

Taking the data collectively, we conclude that the targeting construct introduced into zygotes (along with *Cas9* mRNA and appropriate sgRNAs) has integrated seamlessly at the *Bcl2l11* locus, humanizing the central portion of the gene, as designed.

## Discussion

Contemporary CRISPR technology is revolutionizing genetic engineering and has contributed [along with zinc-finger nuclease (ZFN) and transcription activator-like effector nuclease (TALEN) technologies] to the newly emergent field of gene editing^38,44–56^. The greater CRISPR technique is in a period of rapid expansion, its methodology now being applied across dozens of species^57^ in thousands of laboratories around the globe^58^. Moreover, the seminal core technology continues to diversify with additional enzymatic reagents, novel applications, and technical improvements under robust investigation. This, in turn, has led to a rapid expansion of CRISPR knowledge and the publication of CRISPR reports and reviews on a daily basis.

In the experiments reported here, we set out to explore the utility of using CRISPR technology in mouse zygotes, later brought to term, to replace large (10 s of kbp) segments of the mouse genome with human DNA from orthologous loci. Current CRISPR approaches aimed at knocking experimental DNAs into a locus of interest by homologous recombination have generally involved relatively small genomic expanses from single nucleotides to a few kilobase pairs. Moreover, these experiments routinely make use of long oligonucleotides, or targeting vectors with sub-kilobase homology arms, as donor molecules^59^. Only more rarely are targeting vectors used of the sizes routinely employed in studies involving mouse ESCs^32^.

In contrast to these common practices, we surmised that experimentally altered DNAs, of 10 s to 100 s of kilobase pair lengths, might be directed into a locus of interest if the DNA were outfitted with homology arms 15–30 times longer than those in common use today. Accordingly, we used both ESC and CRISPR Approaches with donor molecules containing 25-kbp of human *BCL2L11* genomic sequence flanked by 15-kbp and 30-kbp mouse homology arms. In addition to success with the ESC/Blastocyst Approach, we have also demonstrated here (by PCR, sequence, and linkage analysis) that replacement of mouse genomic DNA can be achieved using human DNA (of at least 25-kbp) from the corresponding locus and a large vector/CRISPR-stimulated knock-in approach (CRISPR/Zygote Approach).

Given these results, future studies can now begin to explore questions of efficiency and optimization. In our experiment, we performed microinjection into mouse zygotes to see if mice could be recovered, with any degree of humanization of the mouse *Bcl2l11* gene, and if these mice were capable of transmitting the humanized allele through the germline to their offspring. These experiments have demonstrated the utility of this CRISPR/BAC technology to introduce experimental DNA in a directed fashion to the zygotic genome and the ability of the specifically targeted DNA to be transmitted through the germline to progeny. However, due to the small number of data points in whole animal experiments, one can only speculate on the impact of guide selection and donor DNA concentration variables on overall success rates.

Among the experiments in which donor DNA was detected in P_0_ mice (Tables 3 and 4; Experiments 2, 6, and 7), DNA donor concentrations of 1, 5, and 10 ng/µL were represented but the resulting mice show varying degrees of mosaicism. In Experiment 2, where donor DNA concentration was at its lowest (1 ng/µL), donor DNA was not detected among N_1_ progeny (0/50) (Table 5) suggesting that integration of the donor DNA occurred at a multicellular stage of embryonic development and that those cells that did acquire the donor DNA did not contribute to the germline at an appreciable level.

In Experiment 6, where donor DNA concentration was at an intermediate level (5 ng/µL), donor DNA was detected among nearly half of all N_1_ progeny (14/31) suggesting that integration of the donor DNA occurred at the one-cell (zygotic) stage of embryonic development, that that cell gave rise to all cells of the germline, and that the donor DNA was passed, during meiosis, into half of the population of mature germ cells. This result is consistent with our hypothesis that a deletion (ΔJ^+^), of the 17-kbp mouse segment to be replaced, occurred at the *Bcl2l11* locus in the homologous chromosome in the zygote, and was transmitted, in *trans* to the DNA insertion (PJ^+^, DJ^+^), to all remaining progeny (17/17). This result is entirely congruent with the optimal desired outcome, *i*.*e*., where the P_0_ zygote undergoes biallelic modification, develops into a mouse with no mosaicism, and transmits one or the other variant alleles in equal numbers (50%:50%) to the population of mature germ cells.

In Experiment 7, where donor DNA concentration was at the highest level tested (10 ng/µL), the 17-kbp deletion (ΔJ^+^) was detected in only 25% of all N_1_ progeny (14/56), and the donor DNA (PJ^+^, DJ^+^), present in the P_0_ mouse, was not transmitted to the N1 generation at all (0/56). These results can be explained assuming a scenario whereby a deletion occurred in one *Bcl2l11* allele, in a single blastomere, at or near the two-cell stage, and that this deletion-bearing (ΔJ^+^) cell gave rise to roughly half of the developing premeiotic germline and a fourth of all mature (postmeiotic) germ cells. At some later point in blastogenesis, one can hypothesize that an insertion of donor DNA (PJ^+^, DJ^+^) occurred, but in so few cells as to not contribute to the germline in an appreciable way.

A number of aspects in Experiment 7 may have contributed to its less than optimal result. First, due to its viscosity, a donor DNA preparation with a DNA concentration that is too high may not be efficiently delivered through the microinjection needle to the zygote, or delivered in a form less conducive to promoting CAS9 activity and/or HR. Moreover, the guides designed for this experiment, although designed to have an optimal score, did not have what we surmised to be an optimal position, near the ends of the mouse DNA segment to be replaced. It may be that, in experiments of this type, guide position represents a more significant design parameter than guide activity alone.

It is interesting to note that, among all experiments using guides designed for high score optimization, only in Experiment 7, where donor DNA concentration was at the highest level tested (10 ng/µL), was any evidence of donor DNA incorporation (PJ^+^, DJ^+^) seen, and even here it was at a level apparently so low in the P_0_ founder mouse as to not transmit the modified allele to N_1_ mice. You may recall that, in the previously mentioned Experiment 6, where an optimal result was achieved, donor DNA concentration was only 5 ng/µL. It is entirely possible that the successful result seen in that instance was driven by superiorly performing/positioned (nearest the end) guides even at what could prove to be a suboptimal donor DNA concentration. Comparing Experiment 6 with Experiment 7, it is interesting to note that the experiment with the higher donor DNA concentration (Experiment 7, 10 ng/µL) did achieve a higher rate of incorporation (as a percentage of live born mice, 14.3% versus 5.6%) but a lower quality of allele modification in the single founder recovered (mosaicism/transmission of only one modified allele at low frequency compared to nonmosaicism/transmission of both modified alleles at maximum frequency). One may speculate that optimal DNA concentration may be the more important parameter related specifically to the introduction of DNA into individual zygotes; whereas, guide activity may prove to be the more important factor for promoting frequent deletion formation and efficient HR once donor DNA has entered the cell. Experimentation, performed in large numbers of cells, *in vitro*, and further optimization, *in vivo*, are likely to be productive avenues for further research and refinement of this technique.

## Electronic supplementary material

Supplementary Information
