# Supplementary material for: Viable Mice with Extensive Gene Humanization (25-kbp) Created Using Embryonic Stem Cell/Blastocyst and CRISPR/Zygote Injection Approaches
Source: Sci Rep. 2018 Oct 9;8:15028. doi: 10.1038/s41598-018-33408-9 (PMC6177426; doi:10.1038/s41598-018-33408-9)
Supplement: Supplementary file 1 — Supplementary Information [file 41598_2018_33408_MOESM1_ESM.pdf]

SUPPLEMENTARY INFORMATION

Viable Mice with Extensive Gene Humanization (25-kbp) Created Using Embryonic Stem Cell/Blastocyst and CRISPR/Zygote Injection Approaches

Tiffany Leidy-Davis[1], Kai Cheng[1,†], Leslie O. Goodwin[1], Judith L. Morgan[1,‡], Wen Chun Juan[2,§], Xavier Roca[3], S. Tiong Ong[4-7], David E. Bergstrom[1,8,*]

[1]Genetic Resource Science, The Jackson Laboratory, Bar Harbor, ME, USA

[2]Institute of Molecular and Cell Biology, Agency for Science, Technology and Research (A*STAR), Singapore

[3] School of Biological Sciences, Nanyang Technological University, Singapore

[4]Cancer and Stem Cell Biology Signature Research Programme, Duke-NUS Medical School, Singapore

[5]Department of Haematology, Singapore General Hospital, Singapore

[6]Department of Medical Oncology, National Cancer Centre Singapore, Singapore

[7]Department of Medicine, Duke University Medical Center, Durham, NC, USA

[8]Cancer Center, The Jackson Laboratory, Bar Harbor, ME, USA

[†]Current address: Genetically Engineered Models and Services, Charles River Laboratories, Wilmington USA

[‡]Current address: Center for Biometric Analysis, The Jackson Laboratory, Bar Harbor USA

[§]Current address: MSD Pharma (Singapore) Private Limited, Singapore

[*]To whom correspondence should be addressed at dave.bergstrom@jax.org

SUPPLEMENTARY MATERIALS

METHODS

Amplimer Naming Convention

Vector construction made use of numerous small amplimers from human (Supplemental Figure S1.a.1) and mouse (Supplemental Figure S1.a.2) *BCL2L11*- and *Bcl2l11*-containing BACs. For the purposes of the description below, each end of these segments has been given a letter designation corresponding to an oligonucleotide at that end of the amplimer. For example, segment AB is the amplimer arising from amplification with oligonucleotides A and B (Supplementary Table S1).

Bacterial Artificial Chromosome (BAC), Large Plasmid, and Small Plasmid Purification

BACs (100- to 300-kbp) and large plasmids (50- to 100- kbp) were purified using the Qiagen Large Construct Kit (Qiagen, Inc., Germantown, MD USA). Small plasmids (3- to 20-kbp) were purified using Qiagen Plasmid DNA Midi-Prep and Mini-Prep Kits (Qiagen, Inc., Germantown, MD USA).

Plasmid Cloning

Plasmid cloning steps were conducted using common molecular biology reagents obtained from New England BioLabs (Ipswich, MA, USA). Junctional regions were Sanger sequence-verified at each step.

Preparation of the Targeting Vectors/Donor Molecules

Initially, BAC DNAs were purified from BAC clones containing the corresponding *BCL2L11* and *Bcl2l11* genes [human: library RP11, clone 695-B-23; mouse (strain C57BL/6J): library RP23, clone 331-K-22] [1, 2]. Purified DNAs were then electroporated into the recombinogenic *E. coli* strain, SW102 [3].

Segments from the mouse and human BACs were amplified using the oligonucleotides described in Supplementary Table S1, restriction-digested at sites incorporated into the oligonucleotides, gel-purified, and assembled into small plasmid vectors as follows:

Segments KL and MN were cloned along with the neomycin resistance gene- (*Neo*^{R}-) containing *Eco*RI/*Bam*HI fragment of PL452, into a pBluescript II vector (Agilent Technologies, Santa Clara, CA USA) modified to contain an R6Kγ origin of replication [4, 5]. This plasmid is named pTLD01 (Supplemental Figure S1.b.3).

Segments CD, EF, GH, and IJ were cloned along with the neomycin resistance gene- (*Neo*^{R}-) containing *Eco*RI/*Bam*HI fragment of PL451, into a pBluescript II (Agilent Technologies, Santa Clara, CA USA) vector modified to contain an R6Kγ origin of replication [4, 5]. This plasmid is named pTLD02 (Supplemental Figure S1.b.3).

Segments OP, QR, and ST were cloned along with the blasticidin resistance gene- (*Bsd*^{R}-) containing *Eco*RI/*Bam*HI fragment of pTLD08 (a PL452 derivative carrying *att*B, *att*P, and *Bsd*^{R}), into a pBluescript II vector (Agilent Technologies, Santa Clara, CA USA) modified to contain an R6Kγ origin of replication [4, 5]. This plasmid is named pTLD03 (Supplemental Figure S1.b.3).

Segments AB and YZ were cloned into a pBR322-based vector along with the negatively selectable thymidine kinase (*tk*) gene [6, 7]. This plasmid is named pTLD11 (Supplemental Figure S1.b.3).

To begin the assembly of our humanized donor vector proper, pTLD01 was used with standard recombineering approaches to place a *loxP*-flanked neomycin resistance cassette (*Neo*^{R}) just distal to the 2,903-bp deletion region in the human *BCL2L11*-containing BAC [8] (Supplemental Figures S1.c.4 and S1.c.5). After transferring the modified BAC to the *Cre*-expressing *E. coli* strain, SW106, the *Neo* cassette was removed by exposing cells to arabinose (0.1%), leaving a single *loxP* site remaining (Supplemental Figure S1.c.6)[3].

Next, plasmid pTLD02 was used with standard recombineering techniques to place the EF segment of human DNA, a *loxP* site, an FRT-flanked *Neo* cassette, and the GH segment of human DNA just distal to mouse Exon 2 in the mouse *Bcl2l11*-containing BAC (Supplemental Figures S1.d.7 and S1.d.8).

Next, plasmid pTLD03 was used with standard recombineering techniques to place the QR segment of human DNA, and an *attB/attP*-flanked blasticidin resistance (*Bsd*^{R}) cassette, slightly distal to mouse Exon 4 in the pTLD02-modified, mouse *Bcl2l11*-containing BAC described above (Supplemental Figures S1.d.7 and S1.d.8).

Next, plasmid pTLD11 was linearized with *Hind*III and used with standard recombineering procedures to retrieve (*i.e.*, incorporate by recombination) the AB to YZ segment of the mouse *Bcl2l11* gene from the pTLD02/pTLD03-modified BAC (Supplemental Figure S1.e.9), becoming pTLD14 (Supplemental Figure S1.e.10).

At this point, plasmid pTLD14 was purified, digested with *Asc*I, and its two major fragments resolved by agarose gel electrophoresis. The larger of the two linear fragments (Supplemental Figure S1.f.12; approximately 50-kbp) was gel-purified using a QIAEX II Kit (Qiagen, Inc., Germantown, MD USA), and electroporated into recombinogenic *E. coli* cells containing the *loxP*-modified human BAC clone described above (Supplemental Figure S1.f.11), thus capturing the 27,282-bp human segment between flanking mouse homology arms, becoming plasmid pTLD15 (Supplemental Figure S1.g.13).

After experiencing some difficulty with blasticidin-based embryonic stem (ES) cell selection, we replaced the open reading frame (ORF) of *Bsd*^{R} with that of *Puro*^{R} through a negatively selectable *rpsL* intermediate (Supplemental Figure S1.g.14) [9].

This completed vector, pTLD39, performed well in embryonic stem cells subjected to sequential neomycin/puromycin selection.

For the purpose of CRISPR/*Cas9*-based zygotic microinjection, the final *Neo*^{R}/*Bsd*^{R}-containing vector (plasmid pTLD15) was electroporated; first, into the FLP-expressing *E. coli* strain SW105 to remove *Neo*^{R} (making plasmid pTLD66), and next, into a ϕC31 recombinase-expressing *E. coli* strain (an SW105 derivative)

to remove *Bsd*^{R} (Supplemental Figure S1.g.15) [3]. The final vector was named pTLD67 (Supplemental Figure S1.G.16).

Genotyping

Genotyping was performed by routine PCR. Genotyping oligonucleotides are shown in Supplementary Table S2.

RESULTS

Genetic Mapping

Single-nucleotide polymorphism (SNP) genotypes for Chromosome 2 markers of the genetic mapping cross are shown in Supplementary Table S3. Other chromosomes were unlinked to the integration site.

REFERENCES

1. Osoegawa, K., et al., *Bacterial artificial chromosome libraries for mouse sequencing and functional analysis.* Genome Res, 2000. **10**(1): p. 116-28.
2. Osoegawa, K., et al., *A bacterial artificial chromosome library for sequencing the complete human genome.* Genome Res, 2001. **11**(3): p. 483-96.
3. Warming, S., et al., *Simple and highly efficient BAC recombineering using galK selection.* Nucleic Acids Res, 2005. **33**(4): p. e36.
4. Kolter, R., M. Inuzuka, and D.R. Helinski, *Trans-complementation-dependent replication of a low molecular weight origin fragment from plasmid R6K.* Cell, 1978. **15**(4): p. 1199-208.
5. Liu, P., N.A. Jenkins, and N.G. Copeland, *A highly efficient recombineering-based method for generating conditional knockout mutations.* Genome Res, 2003. **13**(3): p. 476-84.
6. Balbas, P., et al., *The plasmid, pBR322.* Biotechnology, 1988. **10**: p. 5-41.
7. Balbas, P., et al., *Plasmid vector pBR322 and its special-purpose derivatives--a review.* Gene, 1986. **50**(1-3): p. 3-40.
8. Copeland, N.G., N.A. Jenkins, and D.L. Court, *Recombineering: a powerful new tool for mouse functional genomics.* Nat Rev Genet, 2001. **2**(10): p. 769-79.

9. Wang, S., et al., *A new positive/negative selection scheme for precise BAC recombineering.* Mol Biotechnol, 2009. **42**(1): p. 110-6.

Abbreviation	Synonym	Orientation	Species	Genome Build	Chromosomal Coordinates	Product Size	Homology Length	Overall Length	Sequence	Enzyme
A	oTLD38	plus	Mouse	mm10	Chr 2 : 128116776 - 128116795	424	20	31	5'-dCGCATACTAGTTCCATCCGGTCATTTCTCTC-3'	*Spe*I
B	oTLD39	minus	Mouse	mm10	Chr 2 : 128117158 - 128117177		20	31	5'-dCGCATAAGCTTTTTTGCTTGGTCCAGATTCC-3'	*Hin*DIII
C	oTLD29new	plus	Mouse	mm10	Chr 2 : 128129327 - 128129348	246	22	35	5'-dCGCATGCGGCCGCATAGTTTAATAACCACCAGGCA-3'	*Not*I
D	oTLD30	minus	Mouse	mm10	Chr 2 : 128129528 - 128129548		21	32	5'-dCGCATAAGCTTAACTGACTGTAGCCCCAGAAA-3'	*Hin*DIII
E	oTLD27	plus	Human	hg38	Chr 2 : 111124898 - 111124917	742	20	31	5'-dCGCATAAGCTTTATTGCTCAGAGGGTTTGGA-3'	*Hin*DIII
F	oTLD28	minus	Human	hg38	Chr 2 : 111125598 - 111125617		20	31	5'-dCGCATGGATCCTGATTTACCTCACTGAAGCC-3'	*Bam*HI
G	oTLD20	plus	Human	hg38	Chr 2 : 111125618 - 111125641	765	24	35	5'-dCGCATGAATTCGGCAGGCCTTTGCCCATGTTATAG-3'	*Eco*RI
H	oTLD21new	minus	Human	hg38	Chr 2 : 111126335 - 111126358		24	37	5'-dCGCATGGCGCGCCCTACTTTACTTCACAGGTATAACC-3'	*Asc*I
I	oTLD31	plus	Mouse	mm10	Chr 2 : 128129549 - 128129573	843	25	38	5'-dCGCATGGCGCGCCGTAGAATTTTCTAAAAACTATATTC-3'	*Asc*I
J	oTLD32	minus	Mouse	mm10	Chr 2 : 128130340 - 128130367		28	39	5'-dCGCATGTCGACGTATTAAGACTCTAATAGCTTCCAGAGG-3'	*Sal*I
K	oTLD9B	plus	Human	hg38	Chr 2 : 111127109 - 111127130	1441	22	35	5'-dCGCATGCGGCCGCTCTCCTTACACTCTGGGAGGAT-3'	*Not*I
L	oTLD10	minus	Human	hg38	Chr 2 : 111128507 - 111128525		19	30	5'-dCGCATGGATCCAACAGCATGATGGTTCCCC-3'	*Bam*HI
M	oTLD11	plus	Human	hg38	Chr 2 : 111128526 - 111128545	501	20	31	5'-dCGCATGAATTCCTCCATAGAGGCTGTGCCAT-3'	*Eco*RI
N	oTLD12	minus	Human	hg38	Chr 2 : 111128985 - 111129004		20	31	5'-dCGCATGTCGACTGAGTGGGAAGAGTCAAGCC-3'	*Sal*I
O	oTLD113	plus	Mouse	mm10	Chr 2 : 128147195 - 128147214	478	20	33	5'-dCGCATGCGGCCGCGTAAGGACCTCTCCCCATCC-3'	*Not*I
P	oTLD114	minus	Mouse	mm10	Chr 2 : 128147618 - 128147646		20	33	5'-dCGCATGGCGCGCCCCAACAGGACAGCCAGCTAC-3'	*Asc*I
Q	oTLD115	plus	Human	hg38	Chr 2 : 111151266 - 111151285	842	20	33	5'-dCGCATGGCGCGCCGTGACTGCTTCCGCTAAAGG-3'	*Asc*I
R	oTLD116	minus	Human	hg38	Chr 2 : 111152064 - 111152083		20	31	5'-dCGCATGAATTCCTCCCCACTTTGATCCTGAA-3'	*Eco*RI
S	oTLD117	plus	Mouse	mm10	Chr 2 : 128147689 - 128147710	510	22	33	5'-dCGCATGGATCCGCATCTTCAGAAGCAGTGTGTT-3'	*Bam*HI
T	oTLD118	minus	Mouse	mm10	Chr 2 : 128148155 - 128148176		22	33	5'-dCGCATGTCGACTCCTCAGTCCATTCATCAACAG-3'	*Sal*I
Y	oTLD40	plus	Mouse	mm10	Chr 2 : 128173955 - 128173974	388	20	31	5'-dCGCATAAGCTTATCAGGCCCAGGGTTCTAGT-3'	*Hin*DIII
Z	oTLD41	minus	Mouse	mm10	Chr 2 : 128174299 - 128174318		20	33	5'-dCGCATGCGGCCGCATAGTGTGCCTGTCCCAAGG-3'	*Not*I

Supplementary Table 1. Oligonucleotides used in the construction of targeting vectors/donor molecules. Intermediate and final targeting vectors/donor molecules were constructed using DNA segments (amplimers) defined by the oligonucleotide pairs above. These segments (amplimers) are designated in the body of this manuscript by the letter designations of the segments' (amplimers') forward and reverse oligonucleotides. For example, Segment AB is an approximately 400-bp sequence amplified from Mouse Chromosome 2 using oligonucleotides A and B above, Segment CD using oligonucleotides C and D above, and so on. Restriction enzyme sites (red) have been incorporated within 11- to 13-base segments of non-homology at the 5' end of each primer. An arbitrary 5'-end sequence (dCGCAT) is shown in blue. See main text, Supplementary Materials text, and Supplementary Figure S1 for more details.

Assay Name	Oligonucleotide Name	Sequence	Length (nt)	Forward/ Reverse	Chromosome	Coordinates (mm10/hg38)	Product Size
**mPJ**	oTLD56	5'-dATCTGTGGCCTTCTAGCCAA-3'	20	Forward	Mouse Chr 2	128129240-128129259	1225 bp
	oTLD57	5'-dAGAATGCCCTAACTCAGCCA-3'	20	Reverse	Mouse Chr 2	128130445-128130464	
**mDJ**	oTLD123	5'-dGTGGACCTCGGTGTGATTTC-3'	20	Forward	Mouse Chr 2	128147042-128147061	760 bp
	oTLD338	5'-dGAGTCAAAGCCTACATCCCCAA-3'	22	Reverse	Mouse Chr 2	128147780-128147801	
**PJ**	oTLD56	5'-dATCTGTGGCCTTCTAGCCAA-3'	20	Forward	Mouse Chr 2	128129240-128129259	773 bp
	oTLD335	5'-dGGAACAGCAAGTCGATCAACAC-3'	22	Reverse	Human Chr 2	111125334-111125355	
**DJ**	oTLD337	5'-dGGTGTTTGAGGAGAGTGCTGTA-3'	22	Forward	Human Chr 2	111151728-111151749	543 bp
	oTLD338	5'-dGAGTCAAAGCCTACATCCCCAA-3'	22	Reverse	Mouse Chr 2	128147780-128147801	
**ΔJ**	oTLD56	5'-dATCTGTGGCCTTCTAGCCAA-3'	20	Forward	Mouse Chr 2	128129240-128129259	713 bp
	oTLD338	5'-dGAGTCAAAGCCTACATCCCCAA-3'	22	Reverse	Mouse Chr 2	128147780-128147801	
**P2.9**	oTLD241	5'-dCCCATCAGAACAGACACTGG-3'	20	Forward	Human Chr 2	111125535-111125554	305 bp
	oTLD236	5'-dTGTAGCTGCTGGGATGTCTG-3'	20	Reverse	Human Chr 2	111125716-111125735	
**D2.9**	oTLD25	5'-dAGCGTAATGTCGTCAGGGTT-3'	20	Forward	Human Chr 2	111128227-111128246	417 bp
	oTLD156	5'-dATGGCACAGCCTCTATGGAG-3'	20	Reverse	Human Chr 2	111128526-111128545	
**Δ2.9**	oTLD241	5'-dCCCATCAGAACAGACACTGG-3'	20	Forward	Human Chr 2	111125535-111125554	207 bp
	oTLD156	5'-dATGGCACAGCCTCTATGGAG-3'	20	Reverse	Human Chr 2	111128526-111128545	

Supplementary Table 2. Genotyping oligonucleotides. Standard PCR primers were designed to amplify the proximal (mPJ) and distal (mDJ) junctions flanking the original mouse *Bcl2l11* allele, the proximal (PJ) and distal (DJ) junctions flanking the humanized *BCL2L11* allele, and the breakpoint (ΔJ) of the deletion-bearing allele. Proximal (P2.9), distal (D2.9), and deletion-spanning (Δ2.9) assays were designed around the 2.9-kbp polymorphism as well. See main text for more details.

SNP	Chromosome-bp	Chr2: 31455512	Chr2: 46607825	Chr2: 62645334	Chr2: 71274980	Chr2: 93981144	Chr2: 113827352	Chr2: 124995482	Chr2: 130144955	Chr2: 159014253	Chr2: 170588407
Positions	RS_ID	rs13476412	rs13476474	rs3022886	rs4223212	rs3682381	rs4223406	rs13476756	rs3662211	rs3689600	rs13476913
Genotypes	C57BL/6J	A	T	G	G	G	T	C	C	G	C
	FVB/NJ	G	C	A	T	T	C	T	A	A	T
	B6FVBF1	A/G	T/C	A/G	T/G	T/G	C/T	C/T	A/C	A/G	T/C
Backcross to FVB/NJ	298-Transgene-carrier	A/G	T/C	A/G	T/G	T/G	C/T	C/T	A/C	A/G	T/C
	301-Transgene-carrier	A/G	T/C	A/G	T/G	T/G	C/T	C/T	A/C	A/G	T/C
	305-Transgene-carrier	A/G	T/C	A/G	T/G	T/G	C/T	C/T	A/C	A/G	T/C
	306-Transgene-carrier	A/G	T/C	A/G	T/G	T/G	C/T	C/T	A/C	A/G	T/C
	307-Transgene-carrier	A/G	T/C	A/G	T/G	T/G	C/T	C/T	A/C	A/G	T/C
	308-Transgene-carrier	A/G	T/C	A/G	T/G	T/G	C/T	C/T	A/C	A/G	T/C
	309-Transgene-carrier	G	C	A	T/G	T/G	C/T	C/T	A/C	A/G	T/C
	310-Transgene-carrier	A/G	T/C	A/G	T/G	T/G	C/T	C/T	A/C	A/G	T/C
	311-Transgene-carrier	A/G	T/C	A/G	T/G	T/G	C/T	C/T	A/C	A/G	T/C
	312-Transgene-carrier	A/G	T/C	A/G	T/G	T/G	C/T	C/T	A/C	A/G	T
	313-Transgene-carrier	A/G	T/C	A/G	T/G	T/G	C/T	C/T	A/C	A/G	T/C
	316-Transgene-carrier	A/G	T/C	A/G	T/G	T/G	C/T	C/T	A/C	A/G	T/C
	317-Transgene-carrier	G	C	A	T/G	T/G	C/T	C/T	A/C	A/G	T/C
	319-Transgene-carrier	G	C	A	T	T	C	C/T	A/C	A/G	T/C
	320-Transgene-carrier	G	T/C	A/G	T/G	T/G	C/T	C/T	A/C	A/G	T/C
	321-Transgene-carrier	G	C	A	T	T	C/T	C/T	A/C	A/G	T/C
	322-Transgene-carrier	A/G	T/C	A/G	T/G	T/G	C/T	C/T	A/C	A/G	T/C
	324-Transgene-carrier	A/G	T/C	A/G	T/G	T/G	C/T	C/T	A/C	A/G	T/C
	326-Transgene-carrier	G	C	A	T	T	C/T	C/T	A/C	A/G	T/C
	303-Wildtype	G	C	A	T	T	C	T	A	A/G	T/C
	304-Wildtype	G	C	A	T	T	C	T	A	A	T
	314-Wildtype	G	C	A	T	T	C	T	A	A	T/C
	315-Wildtype	A/G	T/C	A	T	T	C	T	A	A	T
	318-Wildtype	G	C	A	T	T	C	T	A	A	T
	323-Wildtype	G	C	A	T	T	C	T	A	A	T
	325-Wildtype	G	C	A	T	T	C	T	A	A	T
	327-Wildtype	G	C	A	T	T	C	T	A	A	T
	328-Wildtype	G	C	A	T	T	C	T	A	A	T

SNP	Chromosome-bp	Chr2: 31455512	Chr2: 46607825	Chr2: 62645334	Chr2: 71274980	Chr2: 93981144	Chr2: 113827352	Chr2: 124995482	Chr2: 130144955	Chr2: 159014253	Chr2: 170588407
Positions	RS_ID	rs13476412	rs13476474	rs3022886	rs4223212	rs3682381	rs4223406	rs13476756	rs3662211	rs3689600	rs13476913
Genotypes	C57BL/6J	A	T	G	G	G	T	C	C	G	C
	FVB/NJ	G	C	A	T	T	C	T	A	A	T
	B6FVBF1	A/G	T/C	A/G	T/G	T/G	C/T	C/T	A/C	A/G	T/C
Backcross to C57BL/6J	257-Transgene-carrier	A/G	T/C	A/G	G	G	T	C	C	G	C
	258-Transgene-carrier	A	T	G	G	G	T	C	C	G	T/C
	259-Transgene-carrier	A	T	G	G	G	T	C	C	G	T/C
	260-Transgene-carrier	A/G	T/C	A/G	G	G	T	C	C	G	C
	263-Transgene-carrier	A	T	G	G	G	T	C	C	A/G	T/C
	265-Transgene-carrier	A	T	G	G	G	T	C	C	G	C
	266-Transgene-carrier	A	T	G	G	G	T	C	C	A/G	T/C
	267-Transgene-carrier	A	T	G	G	G	T	C	C	G	C
	270-Transgene-carrier	A	T	G	G	G	T	C	C	G	C
	273-Transgene-carrier	A	T	G	G	G	T	C	C	G	T/C
	275-Transgene-carrier	A/G	T/C	A/G	T/G	G	T	C	C	G	C
	277-Transgene-carrier	A	T	G	G	G	T	C	C	G	C
	261-Wildtype	A/G	T/C	G	G	G	T	C/T	A/C	A/G	T/C
	262-Wildtype	A	T	G	G	G	C/T	C/T	A/C	A/G	T/C
	264-Wildtype	A/G	T/C	A/G	T/G	T/G	C/T	C/T	A/C	A/G	T/C
	268-Wildtype	A/G	T/C	A/G	T/G	T/G	C/T	C/T	A/C	A/G	T/C
	269-Wildtype	A/G	T/C	A/G	T/G	T/G	C/T	C/T	A/C	A/G	T/C
	271-Wildtype	A/G	T/C	A/G	T/G	T/G	C/T	C/T		A/G	T/C
	272-Wildtype	A	T/C	A/G	T/G	T/G	C/T	C/T	A/C	A/G	T/C
	274-Wildtype	A/G	T/C	A/G	T/G	T/G	C/T	C/T	A/C	A/G	T/C
	276-Wildtype	A/G	T/C	A/G	T/G	T/G	C/T	C/T	A/C	A/G	T/C
	278-Wildtype	A	T	G	G	G	C/T	C/T	A/C	A/G	T/C

Supplementary Table 3. Genetic Mapping of the *BCL2L11/ Bcl2l11* Targeting Vector/Donor Molecule Integration Site. Single nucleotide polymorphism (SNP) genotypes for FVB/NJ (Generation P_0), C57BL/6J (Generation P_0), B6FVBF1 (Generation F_1), and fifty mapping progeny (Generation F_2) are shone. The Reference SNP Identification Number (rs_ID) and chromosomal location for each marker are shown across the two uppermost rows. Animal pedigree numbers and targeting vector/donor molecule are shown along the leftmost column (Wildtype = targeting vector/donor molecule is not present). FVB/NJ parental genotypes are shown in blue. C57BL/6J parental genotypes are shown in salmon. Heterozygous/hybrid (F_1) genotypes are shown in green. The critical regions (within which the targeting vector/donor molecule is mapped) are shown for transgene carriers and wildtype mice for each cross (red bordered rectangles). The composite critical interval (within which the targeting vector/donor molecule is mapped) is from markers rs4223406 (Chr2: 113,827,352) to rs3689600 (Chr2: 159,014,253) (red text in column headings) and includes the concordant markers rs13476756 (Chr2: 124,995,482) and rs3662211 (Chr2: 130,144,955) (green text in column headings). See main text for more details.

Experiment Number	Donor Vector (ng/µL)	Guide Set	Zygotes Injected/Transferred	Newborns		Liveborn[1]		Stillborn[2]		Lost Before Wean[3]		Weaned[4]		Founders[5]	
**5**	10	OPTIMAL POSITION (G5-G8)	69	1	1.4%	0	0.0%	1	100.0%	0	N/A	0	N/A	0	N/A
**3**	5	OPTIMAL SCORE (G1-G4)	81	4	4.9%	4	100.0%	0	0.0%	4	100.0%	0	0.0%	0	N/A
**Totals**			**150**	**5**	**3.3%**	**4**	**80.0%**	**1**	**20.0%**	**4**	**100.0%**	**0**	**0.0%**	**0**	**N/A**

[1] Percentage calculated as Liveborn/Newborns X 100
[2] Percentage calculated as Stillborn/Newborns X 100
[3] Percentage calculated as Lost Before Wean/Liveborn X 100
[4] Percentage calculated as Weaned/Liveborn X 100
[5] Percentage calculated as Founders/Weaned X 100

Supplementary Table 4. Failed CRISPR Microinjections. Two microinjection experiments from our CRISPR Approach failed to create wean age mice. Details of these experiments are shown. See main text for more details.

Oligonucleotide Set	Name	Orientation	Species	Genome Build	Chromosomal Coordinates						Product Size	Length	Sequence
**1**	5' Human *BCL2L11*	minus	Human	hg38	Chr	2	:	111125920	-	111125941	222	22	5'd-AGGATGTTATAGTAAGCGAGAA-3'
	5' Human *BCL2L11*	plus	Human	hg38	Chr	2	:	111126121	-	111126141		21	5'd-TAGGTGATGGGTTAATGCATG-3'
**2**	Mouse *Bcl2l11*	minus	Mouse	mm10	Chr	2	:	128150980	-	128150999	351	20	5'd-AACCTAACCCTCGACCCATG-3'
	Mouse *Bcl2l11*	plus	Mouse	mm10	Chr	2	:	128151311	-	128151330		20	5'd-GAGCTGTTCAGGGAGTCAGT-3'
**3**	3' Human *BCL2L11*	minus	Human	hg38	Chr	2	:	111148670	-	111148690	460	21	5'd-TCTGCAGTCTGTGAATAAACC-3'
	3' Human *BCL2L11*	plus	Human	hg38	Chr	2	:	111149109	-	111149129		21	5'd-TTTCCTGTACACGTTACTGAA-3'

Supplementary Table 5. Targeted Locus Amplification (TLA). The precise nature of the targeting vector/donor molecule integration site was determined at single nucleotide resolution using TLA. The three target amplicons used in the studies are described. See main text for more details.

Supplementary Figure S1. Construction of the *BCL2L11/Bcl2l11* Targeting Vectors/Donor Molecules

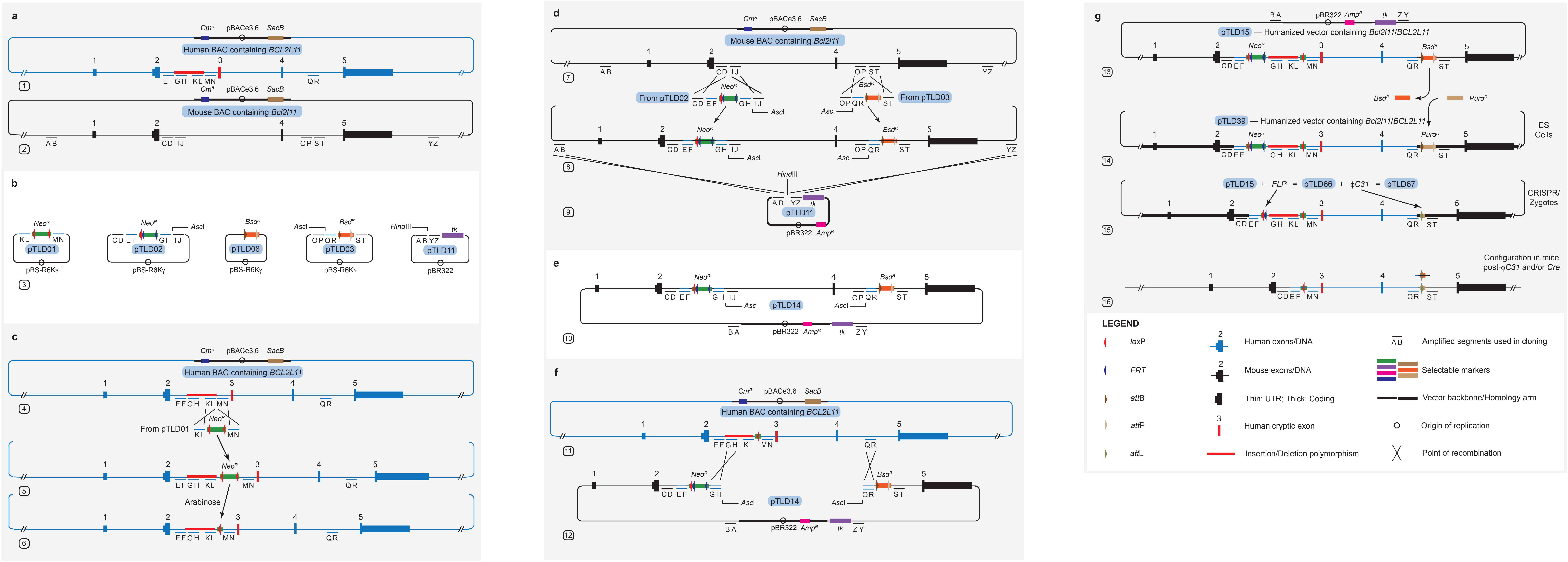

Supplementary Figure 2. Single-Guide RNAs (sgRNAs)

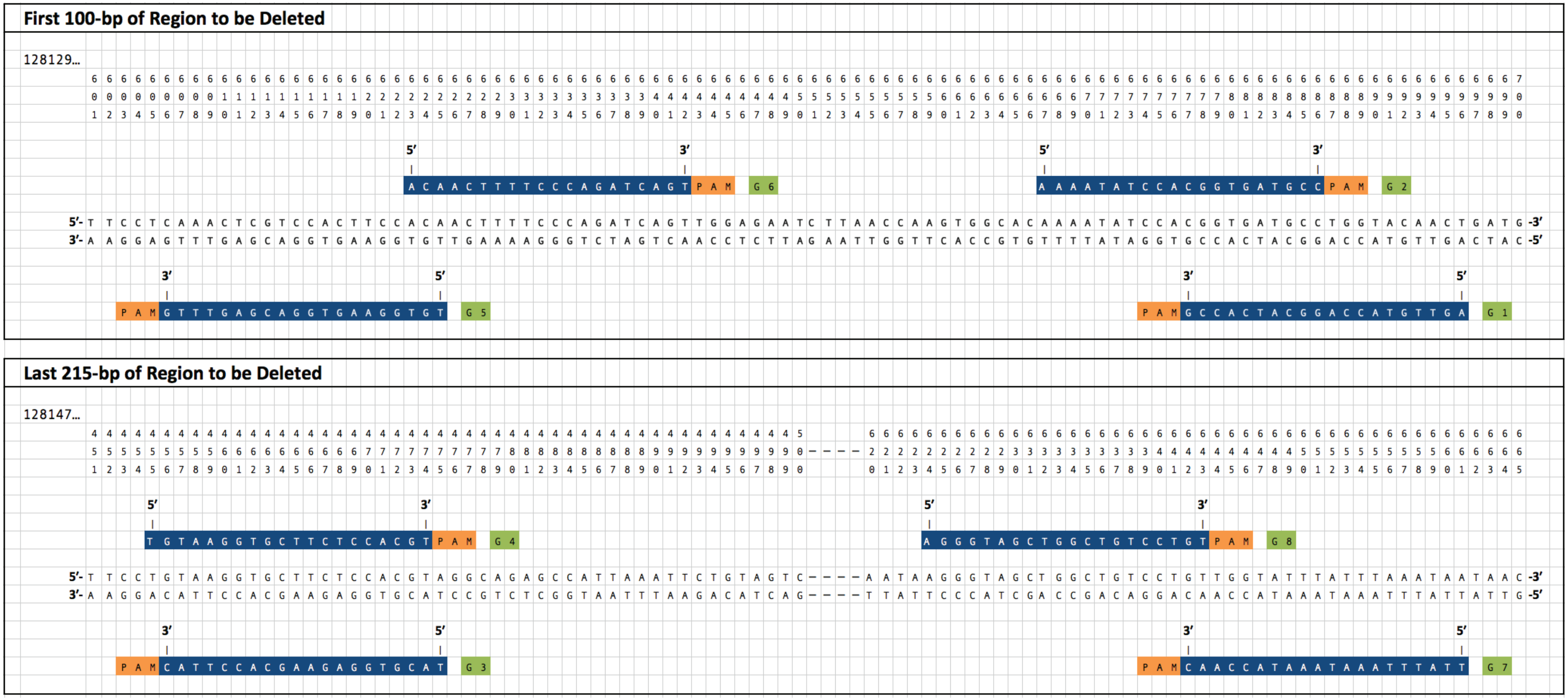

Supplementary Figure Legends

Supplementary Figure S1. Construction of the *Bcl2l11/BCL2L11* targeting vector/donor molecule. A gene-targeting vector/donor molecule was constructed placing a 25-kbp segment of the human *BCL2L11* gene between mouse homology arms, placing removable selectable marker cassettes at each end of the human segment, and placing *loxP* sites around a 2.9-kbp segment of human DNA deleted in 12% of the East Asian population. Final homology arms are shown as thick black lines. See Supplementary Materials text for details.

Supplementary Figure S2. Design and Location of Single-Guide RNAs (sgRNAs). Pairs of sgRNAs were designed along each of two design criteria (optimal scores and optimal positions) at each end (5' and 3') of the mouse central *Bcl2l11* region to be replaced (humanized). Each panel shows the chromosomal (Chr 2) coordinates (mm10) of the base pairs in these regions (upper panel, Chr 2: 128,129,601 – 128,129,700; lower panel, Chr 2: 128,147,451 – 128,147,665) as well as the genomic sequence itself (base pairs are shown from left to right in unshaded cells). The location of each guide is shown above or below the genomic sequence (sgRNA sequences, blue; location of PAM sequence, orange; guide name, green). See main text and Table 1 for more details.
